# Dynamically Modified Flexible Zn Powder Anodes with Stable Performance at High Rate and High Zn Utilization

**DOI:** 10.1002/advs.75967

**Published:** 2026-06-15

**Authors:** Yuxuan Wang, Chenhao Li, Wenbo Zhao, Yong Gao, Yiwei Liu, Yuhao Li, Yu Zhang, Yuhan Yao, Abdelnaby M. Elshahawy, Cao Guan

**Affiliations:** ^1^ Frontiers Science Center for Flexible Electronics Institute of Flexible Electronics Northwestern Polytechnical University Xi'an P. R. China; ^2^ Key laboratory of Flexible Electronics of Zhejiang Province Ningbo Institute of Northwestern Polytechnical University Ningbo P. R. China; ^3^ School of Chemistry Dalian University of Technology Dalian P. R. China; ^4^ Department of Physics Faculty of Science Assiut University Assiut Egypt

**Keywords:** dynamic adjustment, flexible battery, high rate, liquid metal, long‐term stability, Zn powder batteries

## Abstract

Flexible Zn powder‐based batteries hold significant promise for next‐generation portable power‐supply systems. However, their practical applications are hindered by inherent limitations such as poor rechargeability, limited rate capability, and low zinc utilization. Herein, contrary to the widely utilized static modifications on Zn anodes, we integrate a shape‐variant conductive network into the Zn powder electrode, which dynamically homogenizes electric‐field distribution and enhances electron‐transport pathways. The intrinsic fluidity and structural adaptability of the liquid metal‐based network enable effective mitigation of dendritic growth, stress accumulation, and Zn loss during repeated Zn plating‐stripping processes. Consequently, the resulting dynamically shape‐variant electrode exhibits exceptional cycling stability under high‐rate (10 mA cm^−2^/1 mAh cm^−2^ over 1200 h) and high Zn utilization (55.4%, over 400 h and 88.6%, over 244 h), surpassing previously reported Zn powder electrodes. A flexible circuit well integrated with stable NH_4_V_4_O_10_||Zn full‐cell (87.4% capacity retention after 9000 cycles at 10 A g^−1^) is also demonstrated.

## Graphical abstract

A dynamically shape‐variant liquid metal‐based network is integrated into Zn powder electrode, where intrinsic fluidity and structural adaptability of the liquid metal‐based network enable effective mitigation of dendritic growth, alleviation of stress accumulation, and prevention of Zn loss during Zn plating‐stripping processes. As a result, the dynamically responsive electrode exhibits exceptional long‐term cycling stability under high‐rate and high Zn utilization.

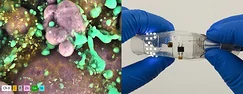

## 1 Introduction

Zinc powder (ZP) anodes exhibit promising potential over Zn foils for commercializing aqueous zinc batteries (AZBs) to achieve the future portable and flexible power supply systems, due to their promising features of low cost, high specific surface area, and large‐scale processibility [1, 2, 3, 4, 5]. However, the large specific surface area of ZP electrodes, while contributing to more uniform nucleation sites, inevitably leads to exaggerated self‐corrosion and continuous Zn loss [6, 7, 8, 9, 10, 11]. In addition, the unevenly distributed electric field seriously affects the stability and reversibility of the repeated Zn plating‐stripping process [12, 13, 14, 15, 16]. These challenges collectively lead to low Coulombic efficiency (CE), inadequate zinc utilization, and poor long‐term cyclability, hindering the practical deployment of ZP electrodes.

To solve the above problems and unleash the potential of ZP electrodes, an effective way is to process the surface of ZP particles with a passivation or shape‐constraint layer. For example, MXene [17], CuO [18], Bi [12], Sn [19], In [20, 21, 22], liquid metal [6] and graphene oxide [23] have been modified onto the surface of ZP particles, which suppress side reactions, uniform electric‐field distribution, adjust plating behavior, and constrain electrode shape‐change, thereby enhancing the cycling stability and CE. Although improved performance has been achieved, the uneven Zn plating‐stripping and shape changes gradually aggregate during cycling, thus only short‐term (< 1000 h) stable performance is achieved at low rates (< 5 mA cm^−2^) with low Zn utilization (< 15%) [24, 25, 26]. In addition, these modification methods rely on static protection, thus the coating layers would be prone to cracking and deterioration during the constant Zn cycling process and different bending/twisting scenarios, which fails for effective protection during long‐term cycles.

Constructing three‐dimensional (3D) structured ZP electrodes [27, 28, 29, 30, 31, 32, 33] and building hydrogel‐based semi‐solid slurry ZP electrodes [25, 34] have also been studied with improved performance. For instance, direct ink writing technology enables the rapid fabrication of 3D Zn electrode frameworks with controlled thickness and geometry [8, 29, 33, 35]. Hydrogels such as polyacrylamide (PAM), polyethylene glycol (PEG), and ethylene vinyl acetate copolymer (EVA) have been explored as alternatives to conventional polyvinylidene fluoride (PVDF) binder to improve hydrophilicity and promote uniform Zn ion distribution [34]. Although these 3D and semi‐solid ZP electrodes could provide certain space to accommodate volume changes through the elastic polymer network, issues of electron‐transport pathways disconnection and nonuniform Zn plating‐stripping behavior still exist [36, 37]. More importantly, such static structural designs also induce lots of unwanted inactive materials, thus long‐term stable cycling under high rate and high depth of discharge (DOD) can be merely achieved [38, 39], and the energy/power density of the electrodes has also been greatly limited. In addition, the complexity of these fabrication methods also impedes the large‐scale production for practical applications. Other reported methods, such as electrolyte additives [40, 41] and facet control [42, 43], also struggle to fundamentally solve the as‐mentioned issues and improve the rate capability, Zn utilization, and cycling stability of ZP electrodes simultaneously. Therefore, it will be of great interest to develop novel strategies that can dynamically uniform electric‐field distribution, maintain electron‐transport pathways, and adjust the Zn plating‐stripping behavior. This will enable high zinc utilization, enhanced high‐rate capability, and prolonged cyclability for a flexible ZP battery.

Herein, contrary to the widely utilized static modification on ZP electrodes, we integrate a dynamically shape‐variant liquid metal network into the flexible ZP film electrode (noted as Zn film with LM), which simultaneously improves cycling stability, high‐rate capability, and zinc utilization ratio. The intrinsic fluidity and variability of the LM enable it to respond dynamically to the local stress and electric field concentration points, effectively homogenizing the zinc plating‐stripping behavior and significantly reducing Zn loss. Concurrently, the LM‐based continuous network significantly enhances the high‐rate capability and long‐term stability under high DOD. As a result, the assembled symmetric cells exhibit remarkable cycling stability and reversibility over 400 h cycling at 55.4% DOD, 244 h cycling at 88.64% DOD, and over 1200 h cycling at a high current density of 10 mA cm^−2^. Paired with NH_4_V_4_O_10_ (NVO) cathode, the full cell exhibits high specific capacity (320 mAh g^−1^) and long‐cycling capacity retention (87.4% after 9000 cycles) under high rate (10 A g^−1^), outperforming previously reported flexible ZP‐based batteries. A flexible electronic system well integrated with stable series NVO||Zn full cells is also demonstrated.

## 2 Results

### 2.1 Schematic Illustration of the Zn Film with LM Anode During Cycling

**FIGURE 1:**
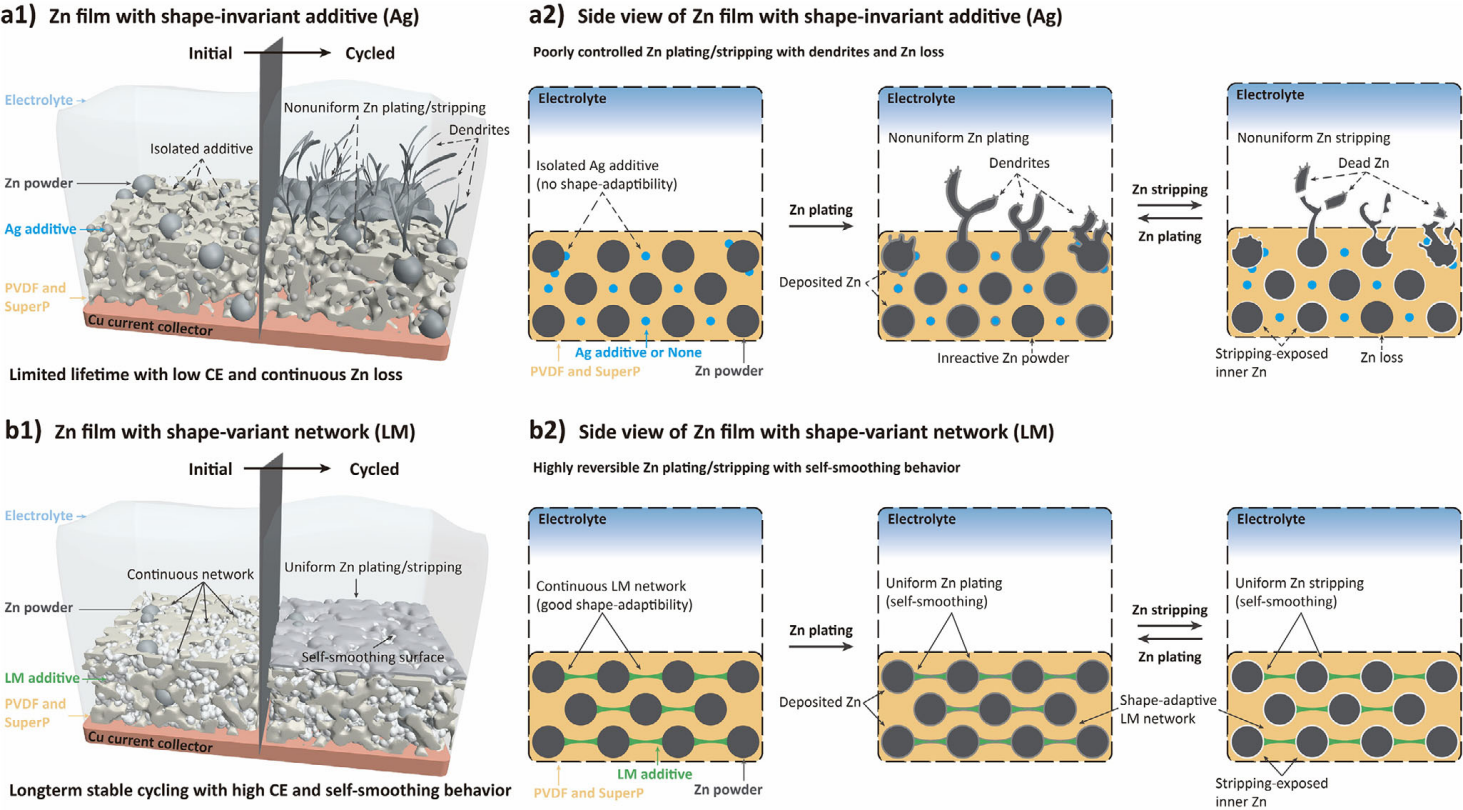
Schematic illustration of the cycling behavior of Zn film electrodes with different additives. (a1‐a2) 3D and side view illustration for Zn film with static shape‐invariant additive (Ag) at initial and cycled states. (b1‐b2) 3D and side view illustration for Zn film with dynamic shape‐variant additive (LM) at initial and cycled states.

Figure 1 shows the schematic comparison of the two different electrodes during cycling. The Zn film with Ag electrode comprises the shape‐invariant Ag nanoparticles inside the electrode (Figure 1a). During cycling, although these isolated Ag additives could promote uniform zinc nucleation by providing abundant zincophilic sites, their agglomeration problem with no shape‐adaptability still easily contributes to a nonuniformly distributed electric field. Therefore, the morphology of the electrode gradually deteriorates with the cycle, leading to continuous dendrite growth and Zn loss. Consequently, the Zn film with Ag electrode shows limited long‐term stability at high rate and high DOD.

As a comparison, the liquid metal (LM) additive is incorporated to form a dynamically shape‐variant network inside the ZP electrode (Figure 1b). Due to its intrinsic fluidity and structural adaptability, the LM additive easily forms an integrally continuous conduction network around Zn powder particles [44, 45]. This network effectively connects ZP particles and enhances electron‐transport pathways, contributing to significantly improved rate capability and minimized inactive isolated Zn. In addition, the LM‐based network could respond to stress and self‐adjust dynamically, which is beneficial for homogenizing the electric‐field distribution and releasing local stress concentration and improving flexible stability. Therefore, the electrode surface exhibits a unique self‐smoothing behavior during the repeated Zn plating‐stripping process, without the formation of dendrites and “Dead Zn”. As a result, the stable long‐term cycling of the flexible ZP electrode under high rate and high DOD conditions would be significantly strengthened.

### 2.2 Fabrication and Physicochemical Characterization

**FIGURE 2:**
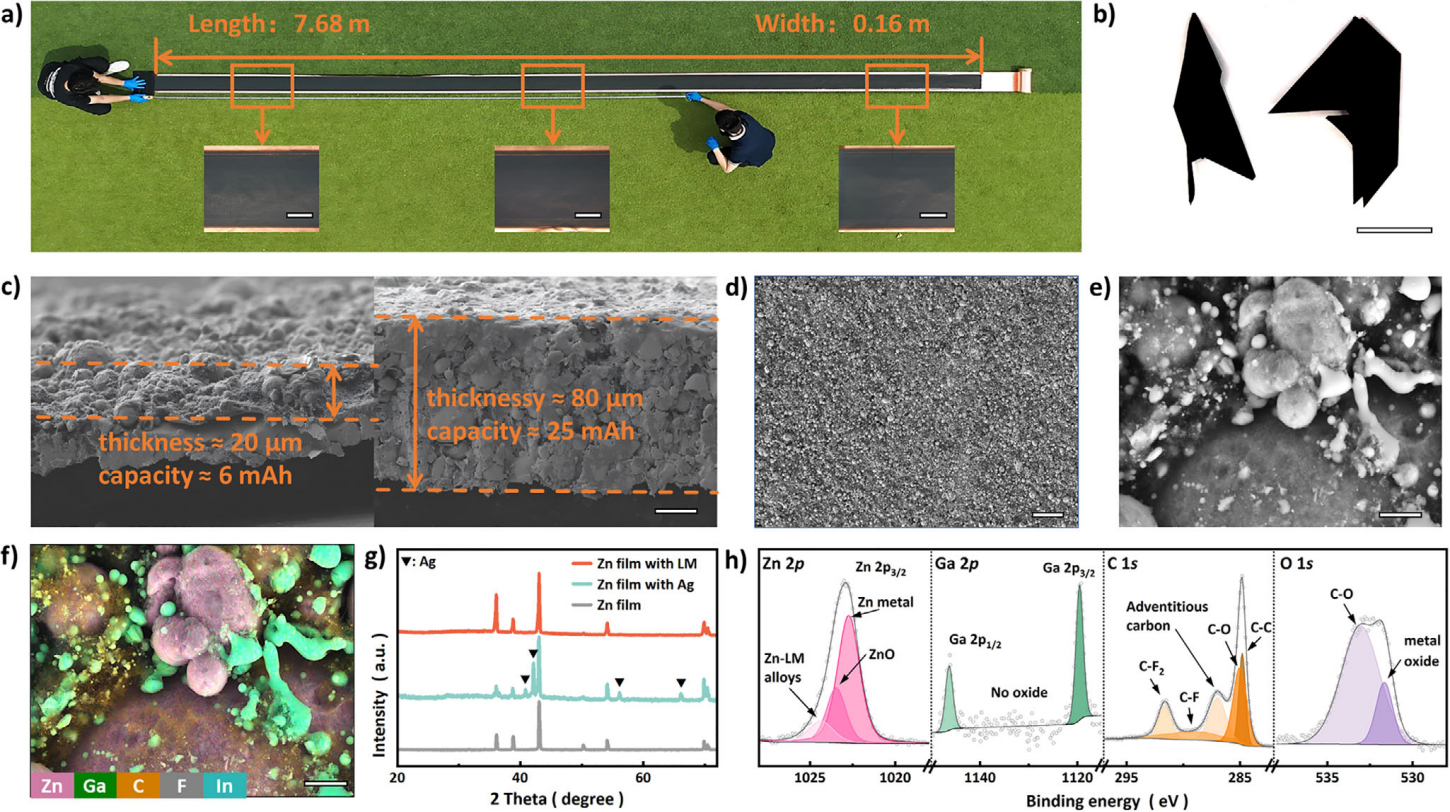
Physicochemical characterization of the Zn film with LM electrode. (a) The large‐scale production and (b) flexibility exhibition of Zn film with LM electrode. (c) SEM side view of prepared Zn film with LM electrodes with low mass loading (20 µm thickness and 6 mAh cm^−2^) and high mass loading (80 µm thickness and 25 mAh cm^−2^). SEM images of the of Zn film with LM electrode (d) small magnification, (e) large magnification and (f) the corresponding EDS mapping results. (g) The XRD patterns of the three different ZP film electrodes. (h) The XPS spectra of Zn 2p_3/2_, Ga 2p, C 1s, and O 1s orbitals at the Zn film with LM electrode. Scale bar: 5 cm for a), 2 cm for b), 20 µm for c), 50 µm for d), 5 µm for e,f).

The Zn film with additives is fabricated by first dispersing the LM or Ag into the 1‐methyl‐2‐pyrrolidinone (NMP) (Figure S1), followed by dissolving PVDF, adding Zn powder, Super P, and casting the film with a final hot‐pressing step (Figure S2, see details in Experimental). This process is highly compatible with commercial production flow, thus enabling large‐scale electrode preparation for practical applications. As a demonstration (Figure 2a), a Zn film with an LM electrode having a width of 0.16 m and a length of 7.68 m is rapidly prepared, where the surfaces in the front, middle, and tail sections are all uniform and smooth. The Zn film with the LM electrode also shows good flexibility and self‐standing property that can be easily folded into the desired shapes (Figure 2b and Figure S2), showing its promising power supply for various flexible electronics. From the side‐view scanning electron microscope (SEM) images in Figure 2c, Zn film with LM electrodes with different thicknesses can be easily adjusted to meet various capacity requirements. From the top‐view (Figure 2d and Figure S3), the surface of the film electrode is extremely smooth with well‐distributed Zn powder and LM network. The microscopic and macroscopic morphology, as well as the thickness of the electrode, can be well controlled in this fabrication process, which fully demonstrates the reliability of this preparation scheme and its potential to be applied in practical applications. From higher magnified SEM images and the corresponding energy dispersive spectroscopy (EDS) mapping results (Figure 2e,f and Figures S4 and S5), majority of the LM forms uniformly interconnected networks and establishes good conductive pathways between Zn powders with a small portion of the LM forming alloys (InGaZn_6_O_9_) on the surface of zinc powder to provide zincophilic sites and introduce Zn plating along (002) plane [46]. In contrast, the electrode using silver powder as the conductive additive is restricted by the intrinsic property of silver powder being prone to agglomeration and unable to flow, resulting in a significantly uneven distribution (Figures S6–S8). A bare Zn film electrode without Ag or LM is also subjected to the same characterizations, where the PVDF binder and conventional conductive Super P fill the gaps between Zn powders (Figures S9–S11). The tensile test of three self‐standing electrodes is performed to verify the macroscopic mechanical properties influenced by Ag and LM additives (Figure S12). The inherent fluidity of LM allows for effective load redistribution under applied stress, thereby preventing localized stress concentration and delaying the onset of fracture, making the electrode more flexible and durable (elongation at break is 8.7%, compared with 3.92% for Zn film with Ag and 1.93% for bare Zn film). This result indicates that the electrode with LM added can avoid local stress concentration caused by high‐capacity and high‐rate Zn plating/stripping, maintain continuous electron and ion conduction pathways, realizing high‐performance ZP electrode with excellent flexibility.

From the X‐ray diffraction (XRD) results (Figure 2g), the characteristic peaks of LM, Ag, and Zn can be clearly identified and are consistent with the standard value. Figure 2h shows the X‐ray photoelectron spectroscopy (XPS) spectra, and one can see the locally formed Zn‐LM alloy, surface ZnO caused by normal air storage conditions, and details of the PVDF binder and Super P additive (Figure S13). The XPS results of the bare Zn film electrode are consistent with those of the Zn film with the LM electrode, except for the peaks of Ga and In (Figure S14).

### 2.3 Simulated Electrochemical Behaviors of Different Electrodes

**FIGURE 3:**
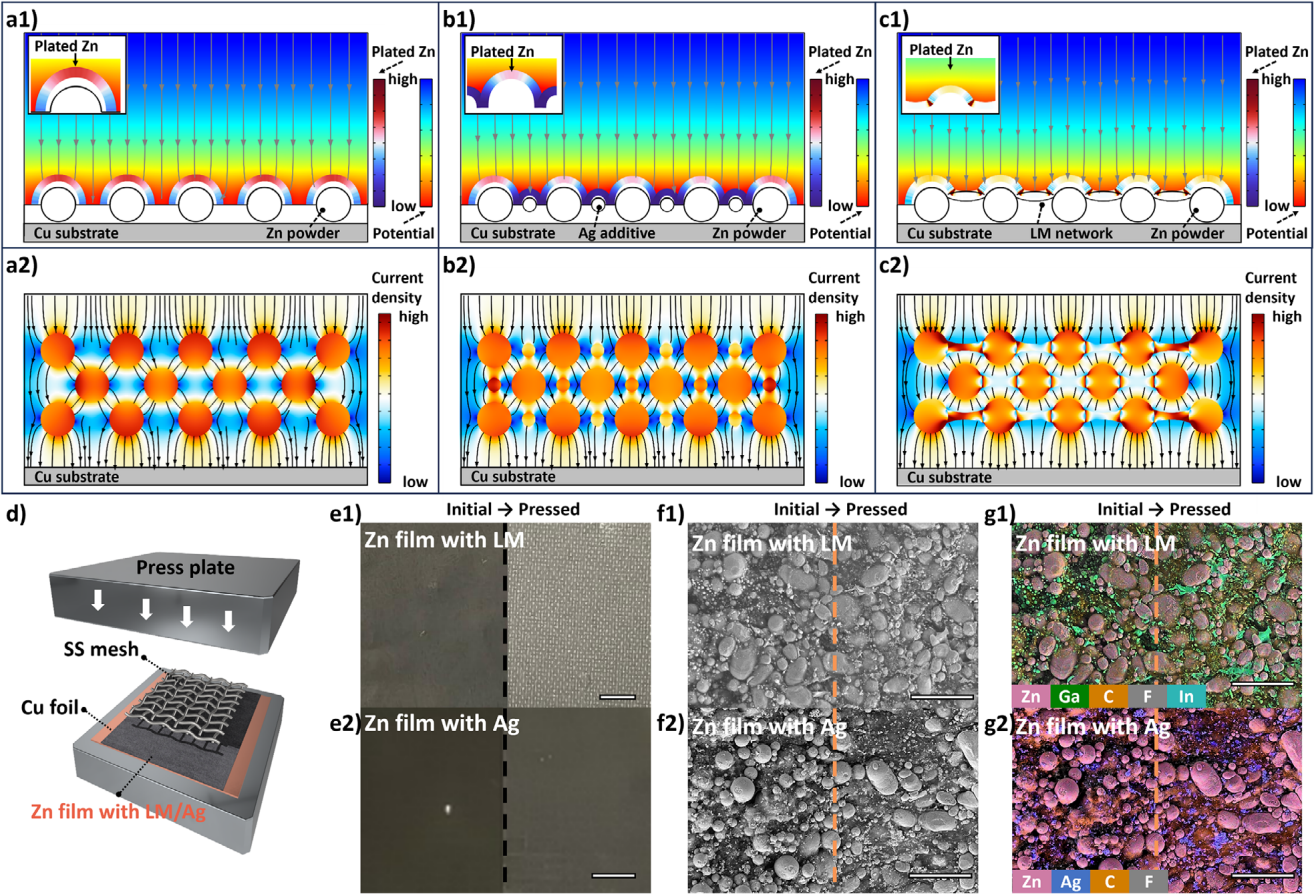
Field simulation and stress characterization. Electric field distribution and Zn plating behaviors for the three different ZP film electrodes: (a1) bare Zn film, (b1) Zn film with Ag and (c1) Zn film with LM. Current density distribution of the three different ZP film electrodes: (a2) bare Zn film, (b2) Zn film with Ag and (c2) Zn film with LM. (d) Schematic diagram of Zn film electrode under local compression. Optical photographs (e) and micro‐level SEM images (f) of Zn film with LM and Zn film with Ag electrodes with (g) the corresponding EDS mappings at the initial state and the pressed state. The LM network has demonstrated obviously dynamic shape‐variant capability. Scale bar:10 µm for e1‐g2.

To further illustrate the advantages of the Zn film with the LM electrode, finite element simulations of Zn plating behavior, electric‐field distribution, and electric conductivity are conducted based on real sample observation (Figure S15). Figure 3a1–c1 illustrates the detailed Zn plating conditions and local electric‐field distributions of three different electrodes. For the bare Zn film electrode (Figure 3a1), due to the potential concentration, abundant Zn is plated at the powder top rather than at the bottom. The “top plating” behavior tends to induce a tip effect, thereby intensifying the dendrite growth and even the short circuits. The introduction of Ag additive brings more nucleation sites, thereby effectively dispersing Zn plating (Figure 3b1). However, isolated Ag additives and ZP particles are unable to establish a stable electrochemical connection, and the issue of top plating persists. This would lead to gradual performance degradation over a certain number of charge–discharge cycles. In contrast, Zn film with LM electrode demonstrates a continuous connection, benefiting from the merits of dynamical shape‐adaptability and fluidity of the LM network (Figure 3c1). As a result, the potential difference between the connective LM network and the top of Zn powders is significantly smaller. Thus, Zn is evenly plated on the whole electrode surface, demonstrating the merits of the self‐smoothing behavior. These simulation differences well demonstrate the excellent morphology control of the Zn film with the LM electrode, even at high‐capacity plating, showing substantial potential to achieve long‐term stable cycling under high capacity and high DOD.

The current density distributions of the three electrodes are further simulated and analyzed (Figure 3a2‐c2). For both bare Zn film and Zn film with Ag electrodes, the current densities are poorly distributed around Zn powders and Ag additives. Consequently, the deteriorated electric field distribution would promote uneven Zn plating and eventually lead to dendrite formation. As a result, the two electrodes show poor cycling reversibility and stability at high rates. On the contrary, the Zn film with the LM electrode shows uniformly distributed electric field intensity within the electrode, which promotes smooth Zn plating between Zn powders (inside the bulk electrode). Therefore, uniform Zn plating in the void space (but not on the top) is effectively realized, which could well accommodate excessive Zn plating and achieve long‐term stable and reversible performance.

The Zn film electrodes with different additives are further pressed using a stainless steel (SS) mesh to simulate their morphological changes under local stress conditions (Figure 3d). From the macroscopic photographs, a distinct difference can be observed. Under localized compression, the Zn film with LM electrode shows a silver–gray dot pattern after pressing (Figure 3e1), indicating the LM network is redistributed, while the Zn film with Ag electrode shows no obvious change of Ag distribution (Figure 3e2). The SEM and EDS are used to reveal the pressed results in detail. For the Zn film with LM electrode (Figure 3f1‐g1 and Figure S16), the initial uniformly dispersed granular LM flows and changes under the local stress, forming a highly structural‐adaptive interconnected network. In contrast, for the Zn film with Ag electrode, the additive remains static with poor point connection (Figure 3f2–g2 and Figure S16). These significant comparisons confirm the dynamic response capability of LM and its unique advantages in constructing a conductive network for a shape‐variant ZP electrode. As a result, problematic issues such as dendrite formation, zinc loss, and stress concentration during the cycling process would be effectively alleviated by the dynamically modified ZP anode, which is expected to significantly enhance the electrode cycling stability at high rates.

### 2.4 Electrochemical Performance and Zn Plating‐Stripping Results

**FIGURE 4:**
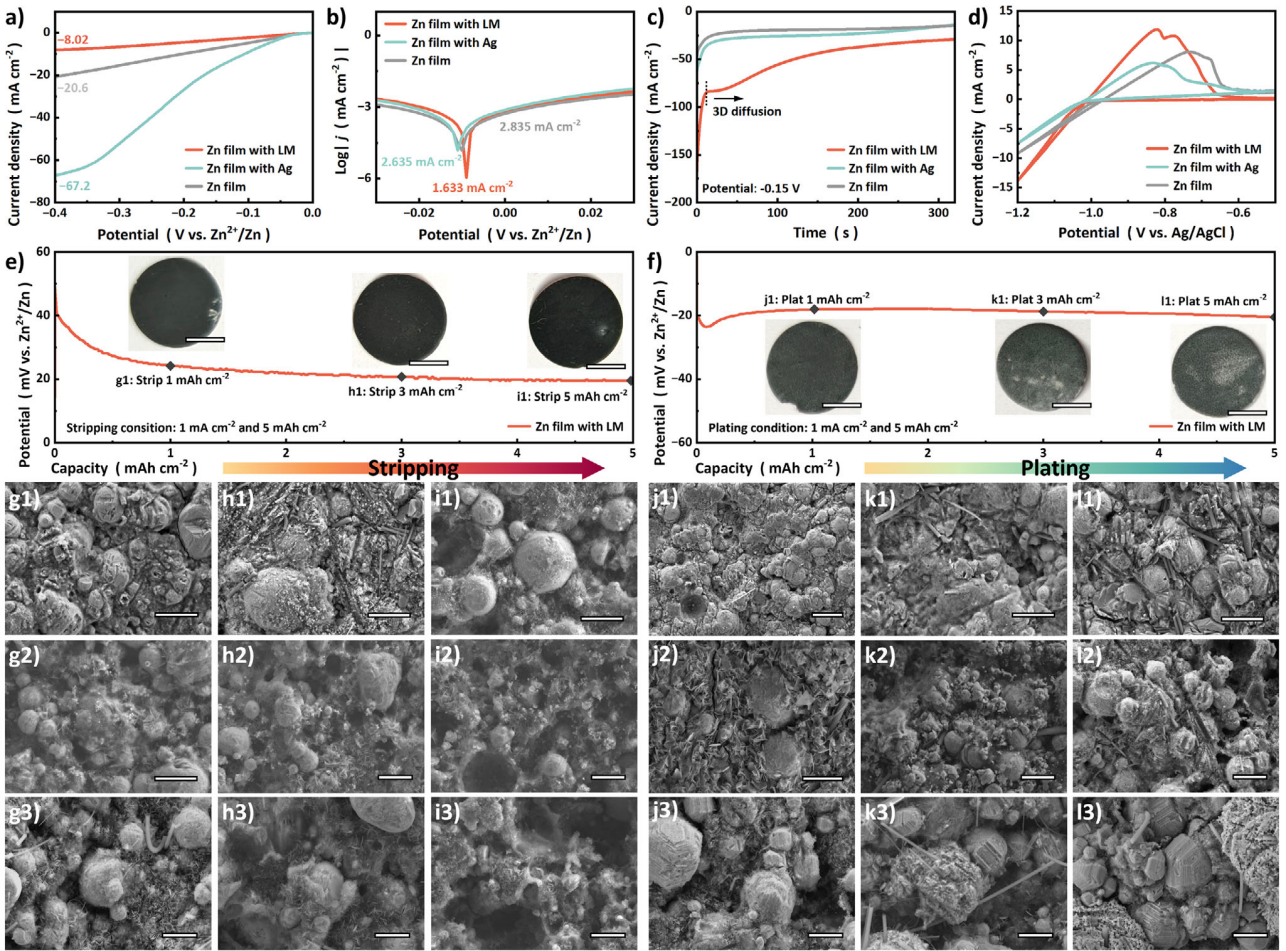
Electrochemical and morphological characterization of the Zn film with LM electrode. (a) HER, (b) Tafel, (c) CA and (d) CV curves of the three different ZP film electrodes. Capacity‐potential profile of Zn film with LM electrode undergoes (e) Zn stripping and (f) Zn plating of 5 mA h cm^−2^ at a constant current density of 1 mA cm^−2^. The insets show the corresponding optical photograph of stripped/plated electrodes. SEM images of Zn film with LM (g1‐l1), Zn film with Ag (g2‐l2), and bare Zn film (g3‐l3) electrodes at the corresponding plating or stripping scenarios. Scale bar: 0.5 cm for (e‐f) insets, and 1 µm for (g1‐l3).

A series of electrochemical tests is carried out to evaluate the electrochemical performance of these ZP film electrodes. From the linear sweep voltammetry (LSV) results in Figure 4a, the Zn film with LM electrode shows the lowest current density of 8.02 mA cm^−2^ at the polarization voltage of −0.4 V, compared with Zn film with Ag (20.6 mA cm^−2^) and bare Zn film (67.2 mA cm^−2^) electrodes, confirming its much–improved resistivity to hydrogen evolution reactions (HER). The Tafel curves (Figure 4b) also show that the Zn film with LM electrode exhibits the lowest corrosion current of 1.63 mA cm^−2^, much lower than that for Zn film with Ag electrode (2.64 mA cm^−2^) and the bare Zn film (2.84 mA cm^−2^), further demonstrating the excellent corrosion resistance. Due to its high zincophilicity, from the chronoamperometry (CA) curves in Figure 4c, the integration of LM network also improves the uniform initial Zn nucleation and subsequent Zn plating processes, thereby benefiting high‐reversible cycling. The cyclic voltammetry (CV) curves of the three electrodes are further tested in a standard three‐electrode system, where Cu foil is used as the counter electrode and Ag/AgCl is used as the reference electrode. From Figure 4d, the Zn film with LM electrode demonstrates smaller voltage hysteresis than that of the Zn film with Ag and bare Zn film electrodes, indicating the accelerated Zn redox kinetics.

To investigate the Zn plating‐stripping results of the three electrodes, Zn with different capacities (1, 3, and 5 mAh cm^−2^) is stripped and plated at a constant current density of 1 mA cm^−2^. From the voltage‐capacity profiles (Figure 4e,f and Figures S17 and S18), during the continuous stripping and plating process, the Zn film with LM electrode demonstrates the minimum overpotential, indicating the lowest plating energy barrier with the highest electrode conductivity. The electrode morphologies are further characterized by optical photographs and SEM images. Through the stripping process. For the Zn film with LM electrode, dynamically shape‐variant LM network well maintains the electron‐conductive pathways, ZP particles could undergo uniform stripping and maintain the original shape even at 55.4% DOD, showing a smooth macro and micro morphologies without apparent dendrites and voids (Figure 4g1‐i1 and Figure S19). In contrast, severe non‐uniform Zn stripping occurs for the other two electrodes, leading to a progressive Zn loss and resulting in poor reversibility in subsequent plating cycles (Figure 4g2–i3 and Figures S17 and S18). During the 5 mAh cm^−2^ plating process. For the Zn film with LM electrode, due to the uniform electric field distribution and the zincophilic alloy (InGaZn_6_O_9_) on the surface of ZP, Zn preferentially nucleates and densely deposits on the ZP particle surface. The voids in the 3D space of this electrode are gradually filled by continuous deposition, indicating a distinctive self‐smoothing plating behavior (Figure 4j1‐l1 and Figure S19). On the contrary, the other two electrodes inevitably fail in local continuous plating with obvious dendrite formation (Figures S17 and S18). The surface morphology differences are significantly distinctive with a higher current density/capacity (10 mA cm^−2^ and 3 mAh cm^−2^) plating and stripping (Figure S20). At such a high rate, the Zn film with LM electrode could still maintain a flat morphology with well‐controlled dense Zn plating‐stripping around the original Zn powder particles, demonstrating self‐smoothing behavior. For the Zn film with Ag electrode, uneven plating/stripping sites can be clearly observed with dendrite formation. The real‐time plating process is also monitored by optical microscopy (Figure S21). Under Zn plating at a high current density of 25 mA cm^−2^, the Zn film with LM electrode, in contrast to the Zn film with Ag electrode, maintained a uniform and compact deposition morphology throughout a 1 h plating period, preserving a smooth surface without the pronounced localized deposition and morphological instability observed on the Ag‐modified electrode. Based on the Zn plating‐stripping results, the dynamically modified ZP anode fully exploits its shape‐variant function in uniform electric field distribution and provides continuous electron pathways, demonstrating its potential for achieving long‐term stable cycling under high rate and high DOD.

### 2.5 Symmetric‐Cell Performance

**FIGURE 5:**
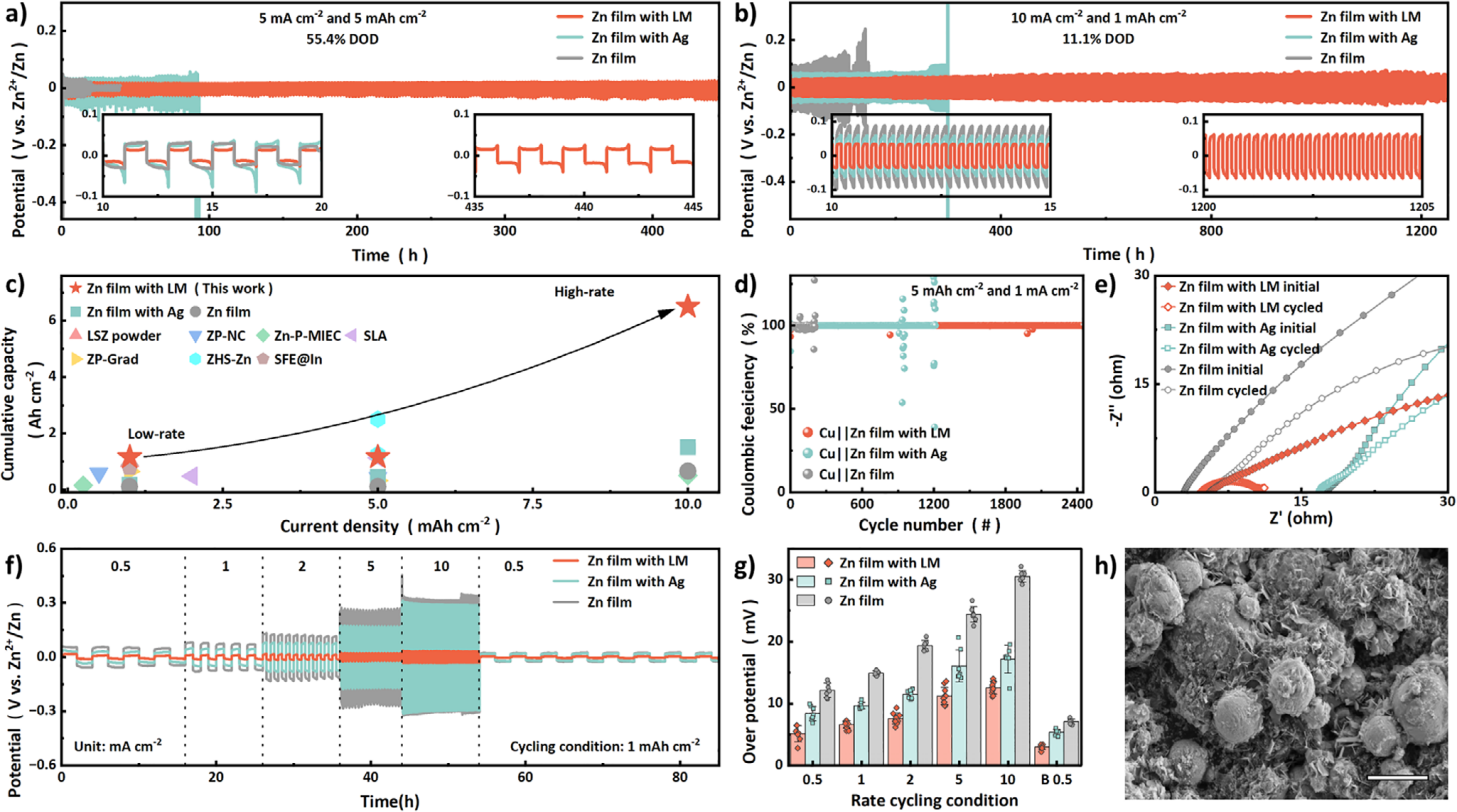
Electrochemical performance of symmetric cells. Voltage profiles of Zn||Zn symmetrical cells with cycling conditions of (a) 55.4% DOD at 5 mA cm^−2^/5 mAh cm^−2^ and (b) 11.1% DOD at 10 mA cm^−2^/1 mAh cm^−2^. (c) Comparison of the Zn film with LM electrode with recently reported Zn powder electrodes and LM‐modified electrodes in terms of cycling current density and cumulative capacity. (d) CE profile for the three ZP film electrodes paired with the Cu foil cathode. (e) EIS plots and fitted curves of the three ZP film cells before and after 10 cycles at 10 mA cm^−2^/1 mAh cm^−2^. (f) Rate performance of the three symmetrical cells and (g) the corresponding nucleation over‐potential comparison at different rate conditions. (h) SEM image of the Zn film with LM electrode after 500 cycles. Scale bar: 10 µm for h.

The addition of this dynamic LM network in the electrode can effectively homogenize the electric field distribution, reduce risks of side reactions, prevent continuous zinc loss and dendrite growth, which is beneficial for long‐term reversible cycling. From the cycling profile in Figure 5a, the Zn film with LM electrode shows stable and low over‐potential cycling over 400 h under high current/high capacity (5 mA cm^−2^/5 mAh cm^−2^) and high Zn utilization (55.4% DOD), exhibiting a much better performance than bare Zn film (short‐circuit at only 25 h) and Zn film with Ag (polarized at only 95 h). Such improvement fully demonstrates the merits of the dynamically modified ZP anode, which maintains electrode conductivity and well controls the high‐capacity plating‐stripping behavior. Even under a higher current density (10 mA cm^−2^ and 1 mAh cm^−2^, Figure 5b), the Zn film with LM electrode still shows an exceptional cycle life exceeding 1200 h, which is a tremendous advantage over bare Zn film (180 h) and Zn film with Ag electrodes (300 h). The above tests confirm that the dynamic LM network design could effectively achieve long‐term stable cycling under varying rate (from 1 to 10 mA cm^−2^), capacity (from 1 to 5 mAh cm^−2^) and DOD (from 11.1 to 55.4%, Figure S22). Even when cycling at 88.64% DOD, Zn film with LM electrode still demonstrates excellent cycling stability and high‐rate stability, achieving stable cycling curves of 52 cycles (over 82 h) under 10 mA cm^−2^/8 mAh cm^−2^ and 61 cycles (over 244 h) under 4 mA cm^−2^/8 mAh cm^−2^ conditions (Figure S23). To note, most previously reported zinc powder anodes (based on static modifications) only performed well at a low current density (< 5 mA cm^−2^) with a limited cumulative capacity (< 2 Ah cm^−2^) and low DOD (< 15%). Therefore, the current density, cumulative capacity, and Zn utilization performance of this Zn film with LM electrode show dominant advantages over those recently reported Zn powder‐based electrodes and LM‐modified electrodes (Figure 5c and Table S1) [3, 6, 15, 16, 27, 34, 35, 38, 43, 46, 47, 48, 49, 50, 51, 52].

Half‐cells using Cu foil as the cathode are further assembled and tested (Figure 5d and Figure S24). Under the high‐rate condition of 5 mA cm^−2^ and 1 mAh cm^−2^, the Zn film with LM electrode achieves an average 99.95% CE over 2500 cycles compared with 98% CE and only 200 cycles for the bare Zn film electrode and 99% CE and 1200 cycles for the Zn film with Ag electrode. This significant distinction highlights the importance of maintaining the conductive path and reducing Zn loss for the Zn powder electrode for the realization of reversible Zn plating‐stripping. The electrochemical impedance spectroscopy (EIS) results of the three ZP film electrodes also indicate the same trends (Figure 5e and Figure S25). After 10 cycles at 10 mA cm^−2^ and 1 mAh cm^−2^, the Zn film with LM electrode exhibits the smallest change in electrochemical impedance, confirming its good electric conductivity maintenance with low charge transfer resistance.

From the rate capability test in Figure 5f, the Zn film with LM electrode shows the best high‐rate charge/discharge performance with the lowest overpotential over this large rate range (0.5–10 mA cm^−2^). The nucleation overpotential results of the rate cycling are further collected and analyzed (Figure 5g). It can be clearly seen that the overpotentials of the Zn film with LM electrode are well maintained at a very low level with good consistency. However, for the other two electrodes, obvious overpotential fluctuations and unstable phenomena occurred when the current density continuously increased. In addition, the surface morphology of the electrode after cycling is also characterized by SEM (Figure 5h and Figure S26). The Zn film with LM electrode still retains a uniform and smooth surface morphology after cycling, confirming the improved cycling stability by the dynamic LM network. The composition of the electrode surface after cycling is analyzed by XPS and XRD (Figures S27–S29), where no obvious by‐products are detected from the Zn film with LM electrode. Together with the post‐cycling SEM images, these results confirm the excellent long‐term stability of this LM‐incorporated electrode.

### 2.6 Full Battery Performance and Integration of Flexible Device

**FIGURE 6:**
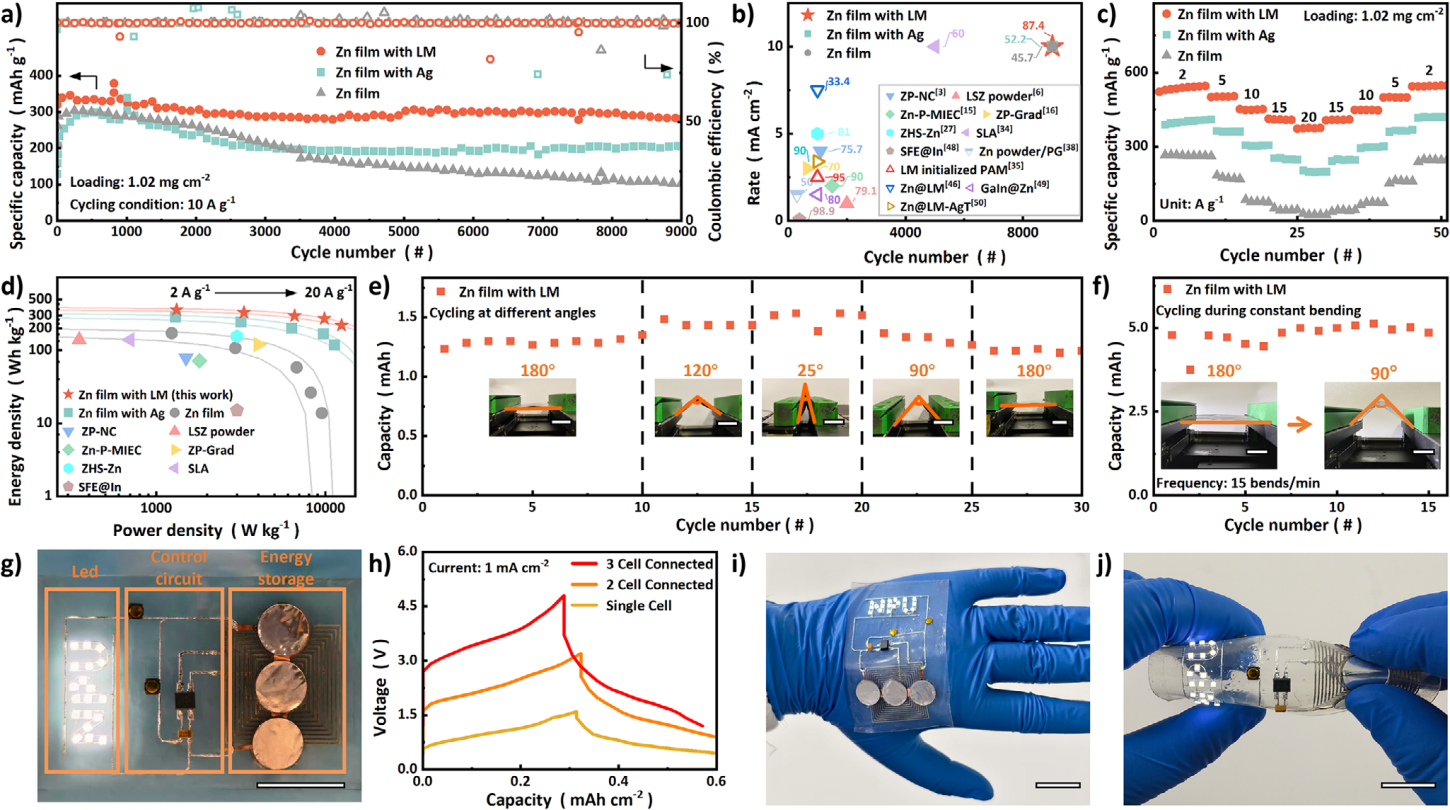
Electrochemical performance of NH_4_V_4_O_10_||Zn full cells. (a) Long‐term cycling performance of assembled full cells using the three different ZP film electrodes. (b) Comparison of the Zn film with LM electrode with recently reported Zn powder electrodes and LM‐modified electrodes in terms of cycle number and current density, the inset numbers indicate the capacity retention values. (c) Rate performance of the three ZP film electrodes‐based full cells and (d) the Ragone plot comparison with recently reported Zn powder‐based full cells. The capacity retention of NH_4_V_4_O_10_||Zn film with LM pouch cells at different scenarios (e) cycling under different angles and (f) cycling during constant bending. (g) A flexible integrated electronic device is assembled and powered by series connected NH_4_V_4_O_10_||Zn film with LM batteries and (h) the corresponding voltage‐capacity profile. Exhibitions of the as‐prepared flexible battery‐integrated electronic device (i) attaching conformally to the back of the hand and (j) working smoothly under large‐scale folding. Scale bar: 2 cm for e, f, g, i and j.

Full batteries using NVO cathode and three different ZP film electrodes are further assembled and tested. In coin‐cells, electrodes of Zn film with conductive additives (Ag or LM) show more protruding oxidation/reduction peaks (Figure S30), indicating faster reaction kinetics, lower internal resistance with higher zinc utilization ratio [47, 53]. From the Nyquist plots (Figure S31), although the LM sample has a higher initial impedance, its dynamics accelerate dramatically after just 10 cycles, emphasizing the advantages of the shape‐variant ability. For the long cycling test (Figure 6a), the Zn film with LM electrode achieves high specific capacity (320 mAh g^−1^) and high‐capacity retention (87.4%) after the constant 9000 cycles (CE > 99.5%). In contrast, for the other two electrodes, obvious capacity attenuation and unstable fluctuations occur during the cycling process, resulting in poor cycling stability. To highlight, the Zn film with LM electrode shows obvious overall advantages compared with previously reported zinc powder anodes and LM‐modified electrodes, in terms of capacity retention and cyclic rate (Figure 6b and Table S2) [3, 6, 15, 16, 27, 34, 35, 38, 43, 46, 47, 48, 49, 50, 51, 52]. The Zn film with LM electrode also shows much‐improved rate capability than the other two electrodes, achieving a high specific capacity of 544 mAh g^−1^ at 2 A g^−1^ and maintaining 370 mAh g^−1^ at a high current density of 20 A g^−1^, indicating a promising rate capability (Figure 6c). In contrast, the Zn film with Ag electrode shows much lower capacity in the tests, and the bare Zn film electrode can hardly be cycled at high current densities. The much‐improved rate capability of the Zn film with LM electrode can be attributed to the dynamically shape‐variant LM network, which effectively homogenizes the Zn plating‐stripping behavior and provides continuous electro‐transport pathways. To note, the NVO||Zn film with LM full cell shows a high energy density of 361.3 Wh kg^−1^ (based on cathode mass) and a high power density of 12.5 kW kg^−1^ (Figure 6d), outperforming previously reported zinc powder anode‐based batteries (Table S2). By appropriately adjusting the mass loadings of cathode (4.2 mg cm^−2^) and anode (5.63 mg cm^−2^), a full cell with a high practical energy density (133.73 Wh kg^−1^, based on total active materials) and a low N/P ratio (negative to positive ratio≈2.2) is also demonstrated (Figure S32). The self‐discharge tests of the full cell further demonstrate that Zn film with LM electrode exhibits good voltage stability and high‐capacity retention during static storage (after 24 h, voltage drop is 0.514 V and the capacity retention of 73.2%), indicating its potential as a portable power source (Figure S33). Based on the above full‐cell tests and the evaluation of material costs (Table S3), the addition of the dynamic LM network has improved zinc powder utilization, optimized rate performance, and enhanced stability and capacity retention at relatively low cost. This dynamically modified electrode achieves a good balance among energy density, cycling stability, mechanical flexibility, and cost‐effectiveness under typical conductive additive usage, indicating great potential for practical applications.

A series of pouch cells of different sizes is assembled and subjected to flexible cycling tests under different bending conditions (Figure S34). From Figure 6e, the NVO||Zn film with LM pouch cell shows steady capacity even when bent to 25 degrees, which is in an almost folded state. The excellent flexibility is further confirmed by the EIS measurements, which reveal very small variation between the initial state and after ten bending cycles (Figure S35). From Figure 6f, a more challenging dynamic bending test is further carried out by bending the pouch cell at a frequency of 15 bends per minute from 180 degrees to 90 degrees. As expected, the dynamically modified LM‐based cell demonstrates excellent stability and unaffected high capacity upon continuous bending. The optical photographs (Figure S30b2) of the electrodes obtained from the disassembled pouch cell further indicate that the Zn film with LM electrode can maintain its original state and remains in good condition after undergoing bending‐cycling tests.

As a further demonstration, a flexible integrated device is constructed on a soft elastomer substrate with LM‐based connecting wires, in which functional devices such as NVO||Zn film with LM battery, light emitting diode (LED) tubes (2.6–2.8 V), wireless charging coils, and the corresponding control circuit are well integrated (Figure 6g, Figure S36, and Movie S1). The NVO||Zn film with LM battery functioning in series can well meet the power supply requirements and light up the LEDs marked “NPU”. From Figure 6h, the capacity‐voltage profile confirms that the voltage output is almost three times that of a single battery. In addition, this flexible battery‐integrated device can be conformally attached to the back of the hand (Figure 6i) and maintain its function even under a large extent of bending (Figure 6j and Movie S2), confirming the promising potential of Zn film with LM electrode working as the power supply for various flexible electronics.

## 3 Conclusions

In summary, contrary to the widely utilized static modification on Zn anodes, we have constructed a shape‐variant LM network for flexible ZP electrode that dynamically adjusts the cycling behavior. The intrinsic fluidity and structural adaptability of LM provide uniformly distributed electric‐fields and continuous electro‐pathways, effectively suppressing dendrite growth, stress concentration, and continuous zinc loss. As a result, the dynamically shape‐variant flexible ZP electrode achieved stable cycling over 1200 h at 10 mA cm^−2^, 400 h at 5 mA cm^−2^/5 mAh cm^−2^/55.4% DOD, and 244 h at 4 mA cm^−2^/8 mAh cm^−2^/88.6% DOD, surpassing previously reported Zn‐powder electrodes. Full cells also illustrate high‐rate capability (370 mAh g^−1^ at 20 A g^−1^) and high‐capacity retention with long life‐time (87.4% after 9000 cycles). A battery‐integrated flexible device with excellent bending performance is also demonstrated. The large‐scale, stable, high‐rate, and high DOD Zn powder batteries provide a promising strategy for future portable power supply.

## 4 Method

### 4.1 Zinc Anode Preparation

The Zn film with LM electrode is fabricated through a three‐step process: slurry preparation, coating, and hot‐press (Figure S2a). First, a homogeneous suspension was obtained by dispersing 1.0 g of LM (HuaTai Metal Materials) in 15 mL of NMP (Adamas) via probe sonication (70% amplitude, 0°C, 10 min). Subsequently, 0.70 g of PVDF (Canrd Co., Ltd.), 8.40 g of Zn powder (Guangdong Guanghua Sci‐Tech Co., Ltd.), and 0.35 g of Super P (Canrd Co., Ltd.) were sequentially added to the suspension. The mixture was then stirred overnight at 40°C to form a uniform slurry (Figure S37), and the approximate mass ratio of the electrodes is Zn powder: LM: Super P: PVDF ≈ 80.38: 9.6: 3.35: 6.7. The slurry was then coated onto a 10 µm‐thick copper foil (Kejing Corp. Ltd) using a doctor blade (Kejin Corp. Ltd). After resting for 30 min, the sample was dried overnight in a 60°C oven. The dried electrode film was subsequently compacted by hot‐pressing at 25 MPa and 40°C for 15 min, during which the LM formed a continuous conductive network. Zn films with thicknesses ranging from 20 to 80 µm were readily fabricated; a 40 µm‐thick film with active material loading of 11 mg cm^−2^ was selected for further electrochemical testing, unless otherwise stated. Finally, the electrode was punched into 12 mm disks for cell assembly and testing.

The Zn film with Ag electrode was prepared following the same procedure, except using 1.40 g of fine silver powder (Adamas), and the approximate mass ratio of the electrodes is Zn powder: Ag powder: Super P: PVDF ≈77.42: 12.9: 3.23: 6.45.

The bare Zn film electrode was fabricated without any additives, and the approximate mass ratio of the electrodes is Zn powder: Super P: PVDF ≈88.89: 3.7: 7.41.

### 4.2 NH_4_V_4_O_10_ Cathodes Preparation

The NH_4_V_4_O_10_ cathode material was synthesized via a hydrothermal method. Initially, 0.64 g of ammonium vanadate (Macklin) was dissolved in 80 mL of deionized water under stirring at 60°C for 10 min. Then, 1.16 g of oxalic acid dihydrate (Adamas) was added, and stirring continued for 40 min until a dark‐green solution formed. This solution was transferred into a Teflon‐lined autoclave and reacted at 180°C for 24 h. After cooling to room temperature, the resulting precipitate was collected, washed repeatedly with DI water and ethanol, and dried at 60°C under vacuum for 24 h to obtain NH_4_V_4_O_10_ powder.

The cathode slurry was prepared by mixing the synthesized NH_4_V_4_O_10_ powder, Super P, and PVDF in a mass ratio of 7:2:1 via ball milling (ChiShun Tech.). Based on PVDF usage, an appropriate amount of NMP was added, and the mixture was milled for 24 h to achieve homogeneity. The resulting slurry was coated onto a 10 µm‐thick titanium foil (Canrd Co., Ltd.) using a doctor blade, followed by drying under vacuum at 60°C for 6 h. The final electrode disks (12 mm diameter, Figure S38) were punched from the dried film, with an active material loading of approximately 1.02 mg cm^−2^ unless otherwise stated.

### 4.3 Materials Characterization

Field emission scanning electron microscopy (FE‐SEM) studies were conducted using Gemini SEM 300 (30 kV, ZEISS) equipped with an Energy dispersive spectrometer (EDS) (Thermofisher, Thermo NS7). X‐ray diffraction (XRD) patterns were obtained from Bruker D8 Advance with radiation from a Cu target. The X‐ray photoelectron spectroscopy (XPS) spectra were obtained from Kratos (Axis Supra). And the bending tests were carried out based on a flexible materials and device testing system (FlexTest, NanoUPE).

### 4.4 Finite Element Simulation

The simulation was carried out using COMSOL Multiphysics software version 6.3. Based on the electrode structure (Figure S14), the top Zn plating behavior was simulated by applying a transient 2D electrodeposition model to study the electric field distribution and continuous Zn plating results. The current density distribution and conduction pathways were carried out by applying a current terminal of 5 mA cm^−2^ between the top and bottom boundaries of the electrodes in the AC/DC module. The initial boundary condition, electric conductivity, and ionic conductivity data were set based on the used Zn, Ag, and LM materials and 2 m ZnSO_4_ electrolyte. The half‐cell voltage hysteresis was set as the cathodic potential, and the anodic potential was 0 V.

### 4.5 Electrochemical Measurement

For the electrochemical performance of the electrodes, CR‐2025‐type coin cells (Neware) were assembled using a glass fiber separator (GF‐A, Whatman) and 80 µL 2 m ZnSO_4_ electrolyte. Half‐cells were assembled using a Cu foil (20 µm thickness, 12 mm diameter) as the cathode, and the upper cut‐off voltage was set at 1 V (vs. Zn^2+^/Zn). Full cells were assembled by pairing the prepared NVO cathode with the Zn film electrodes, using approximately 80 µL of 3 m Zinc trifluoromethanesulfonate (Zn(OTf)_2_, Sigma–Aldrich) as the electrolyte to better match the as‐prepared vanadium‐based cathode. These full‐cells were operated within a voltage window of 0.4–1.6 V without any pre‐cycling. Single‐layer pouch cells were also assembled using differently sized cathodes and anodes, about 100 µL of electrolyte, 38 µm thick aluminum‐plastic film encapsulation, and aluminum/nickel tabs. All the cells were galvanostatically cycled using a Land battery testing system (CT3002A) after a resting process. And the LSV, Tafel, CA, CV, and EIS measurements were conducted by using the electrochemical workstation (CHI760e, China).

### 4.6 Calculations

The areal capacity of the electrode is calculated: Areal capacity (mAh cm^−2^) = Areal mass loading (mg cm^−2^) × Theoretical capacity of Zn (mAh g^−1^).

For 20 um thickness film: 9.1 (mg cm^−2^) × 80.4% × 820 (mAh g^−1^) = 6 (mAh cm^−2^).

For 80 um thickness film: 30.5 (mg cm^−2^) × 80.4% × 820 (mAh g^−1^) = 25 (mAh cm^−2^).

The Zn utilization and DOD were calculated accordingly.

Zn utilization = DOD = Discharge capacity / Theoretical areal capacity * 100% = Cycled capacity / (Zn powder loading * 820 mAh g^−1^) * 100%

The full‐cell energy and power density were calculated based on the active material in the cathode and anode.

Energy density (Wh/kg) = Discharge platform voltage (V) x Discharged capacity (mAh) ÷ (cathode material mass + anode material mass).

Power density (W/kg) = Discharge platform voltage (V) x Current density (mA) ÷ (cathode material mass + anode material mass).

### 4.7 Flexible Electronic Device

A rectangular piece of an acrylic‐based UV‐curable elastomer was used as the flexible substrate for the wireless charging energy system. LM was patterned onto this substrate to form the conductive circuits. Subsequently, a flexible battery array, a rectifier, a capacitor, a resistor, and an LED array (2.6–2.8 V) were assembled in sequence onto the patterned substrate. After assembly, the components were encapsulated using the same elastomer to yield a fully integrated flexible electronic device.

## Author Contributions

C. G. directed and led the research. Y. W. performed research and wrote the paper. C. L. and Y. L. prepared samples and assembled the cells. Y. W. and Y. G. performed the COMSOL simulations. Y. L., Y. Z., A.E., and Y. Y. analyzed data. And W. Z. assembled the flexible device.

## Conflicts of Interest

The authors declare no conflict of interest.

## Supporting information




**Supporting File 1**: advs75967‐sup‐0001‐SuppMat.docx.

**Supporting File 2**: advs75967‐sup‐0002‐MovieS1.GIF.

**Supporting File 3**: advs75967‐sup‐0003‐MovieS2.GIF.

## Data Availability

The data that support the findings of this study are available from the corresponding author upon reasonable request.
