# Supplementary material for: Dynamically Modified Flexible Zn Powder Anodes with Stable Performance at High Rate and High Zn Utilization
Source: Adv Sci (Weinh). 2026 Jun 15:e75967. Online ahead of print. doi: 10.1002/advs.75967 (PMC13336622; doi:10.1002/advs.75967)
Supplement: Supplementary file 1 — Supporting File 1: advs75967‐sup‐0001‐SuppMat.docx. [file ADVS-9999-e75967-s003.docx]

**Supplementary Information**

**Dynamically Modified Flexible Zn Powder Anodes with Stable Performance at High Rate and High Zn Utilization**

*Yuxuan Wang^1,2,5^, Chenhao Li^1,2,5^, Wenbo Zhao^1^, Yong Gao^3^, Yiwei Liu^1,2^, Yuhao Li^1^, Yu Zhang^1^,* *Yuhan Yao^1^, Abdelnaby M. Elshahawy^1,4^, and Cao Guan^1,2*^*

^1^Frontiers Science Center for Flexible Electronics, Institute of Flexible Electronics, Northwestern Polytechnical University, Xi’an 710072, P. R. China.

^2^Key laboratory of Flexible Electronics of Zhejiang Province, Ningbo Institute of Northwestern Polytechnical University, 218 Qingyi Road, Ningbo, 315103, China.

^3^School of Chemistry, Dalian University of Technology, Dalian 116024, China.

^4^Department of Physics, Faculty of Science, Assiut University, Assiut, Egypt.

^5^These authors contributed equally: Yuxuan Wang, Chenhao Li.

Corresponding Author

^*^E-mail: iamcguan@nwpu.edu.cn (C. Guan)

Keywords: Zn powder batteries, dynamic adjustment, liquid metal, long-term stability, high rate, flexible battery


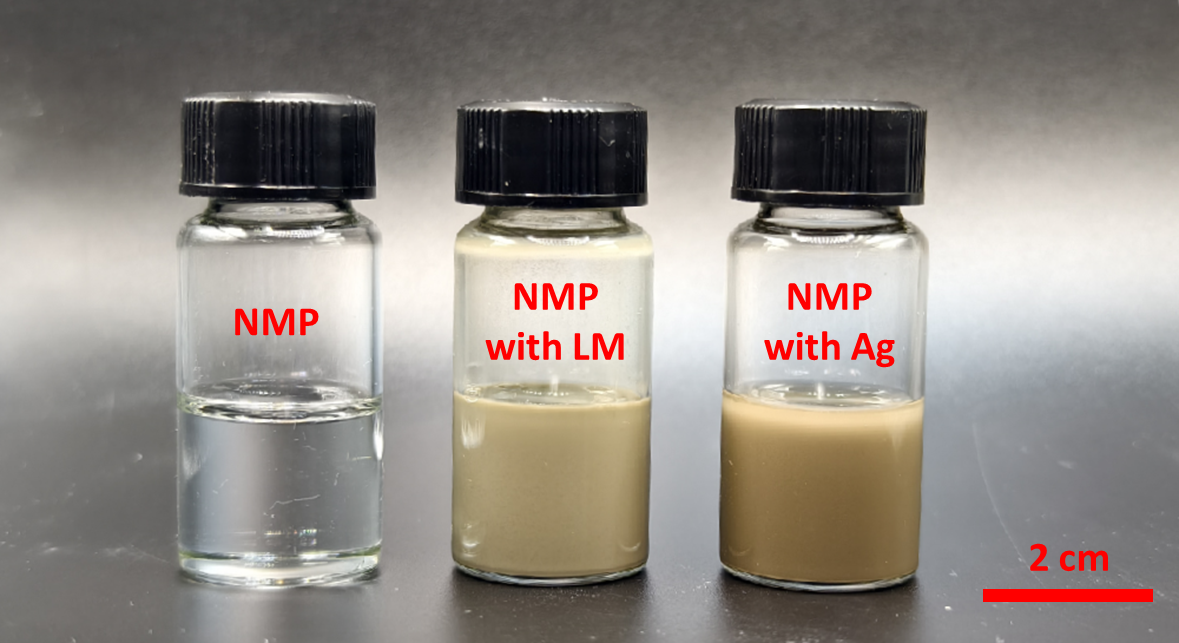


**Figure S1.** The additive dispersions: Pure solvent, NMP with LM dispersion and NMP with Ag dispersion.


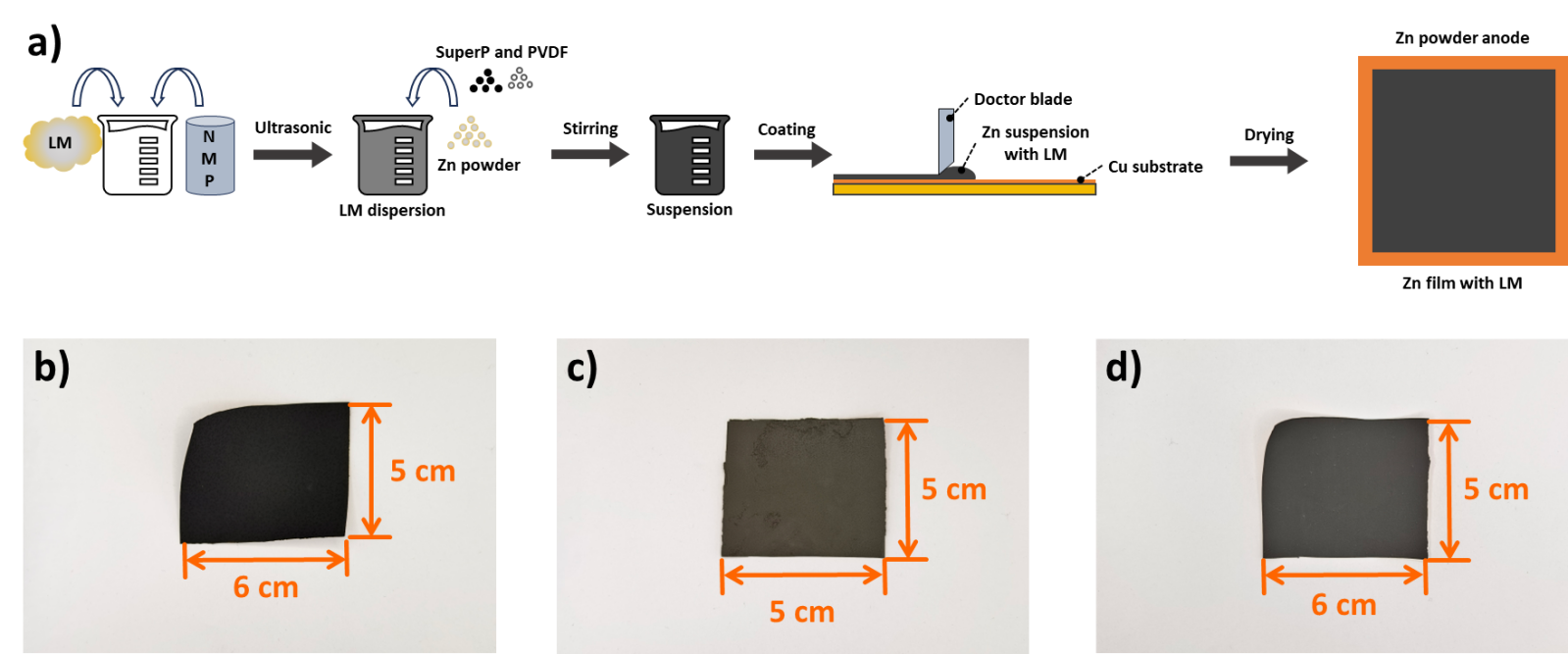


**Figure S2**. (a) Schematic Illustration of the Zn film with LM electrode preparation procedures. Optical photographs of three different electrodes (b) bare Zn film, (c) Zn film with Ag, and (d) Zn film with LM electrode.


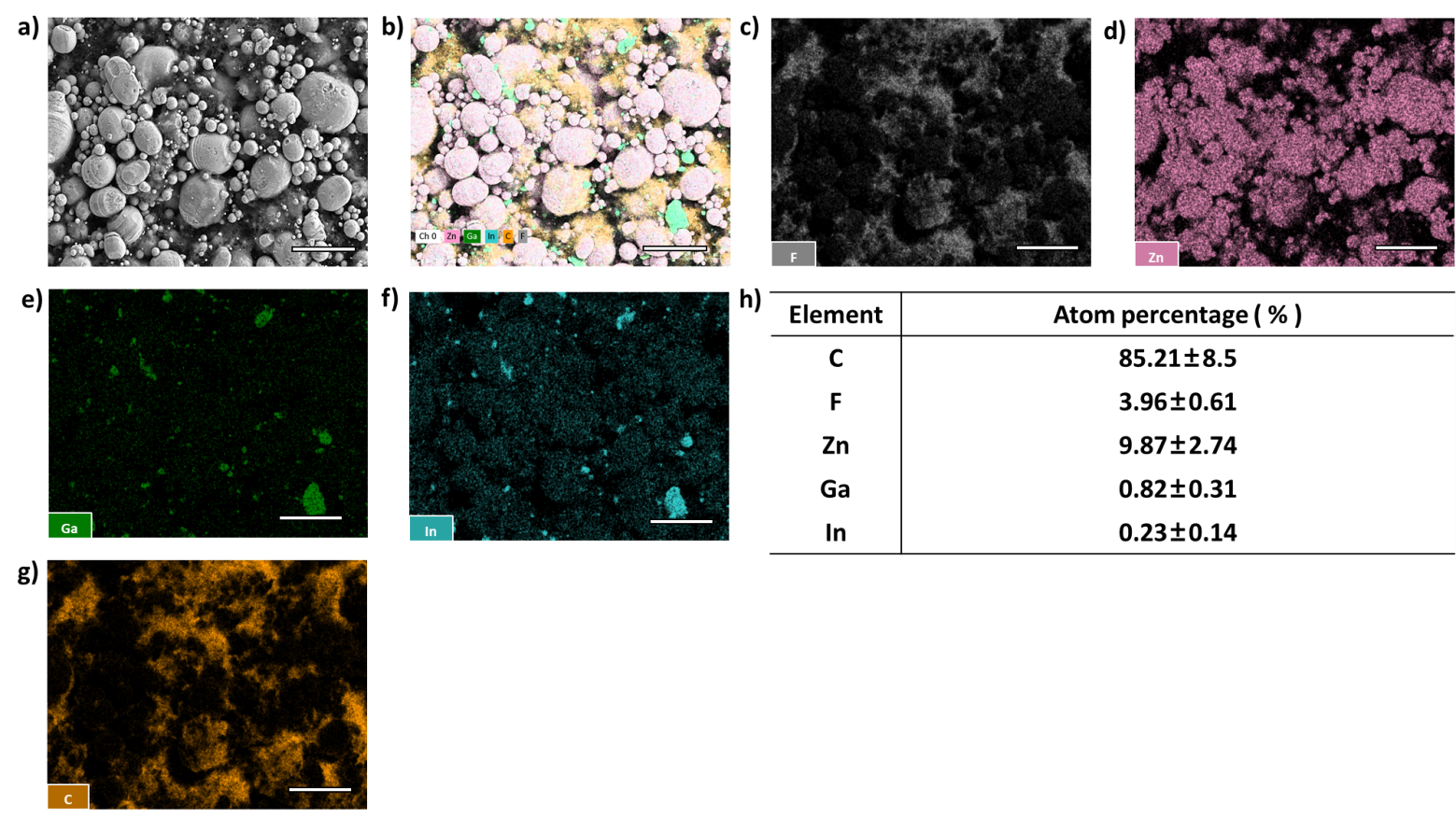


**Figure S3.** Low-magnification morphology and elemental characterization of Zn film with LM electrode. (a) SEM image. EDS mapping results of (b) all, (c) F, (d) Zn, (e) Ga, (f) In, (g) C, and the atom percentage table (h) corresponding to b. Scale bar: 20 μm.


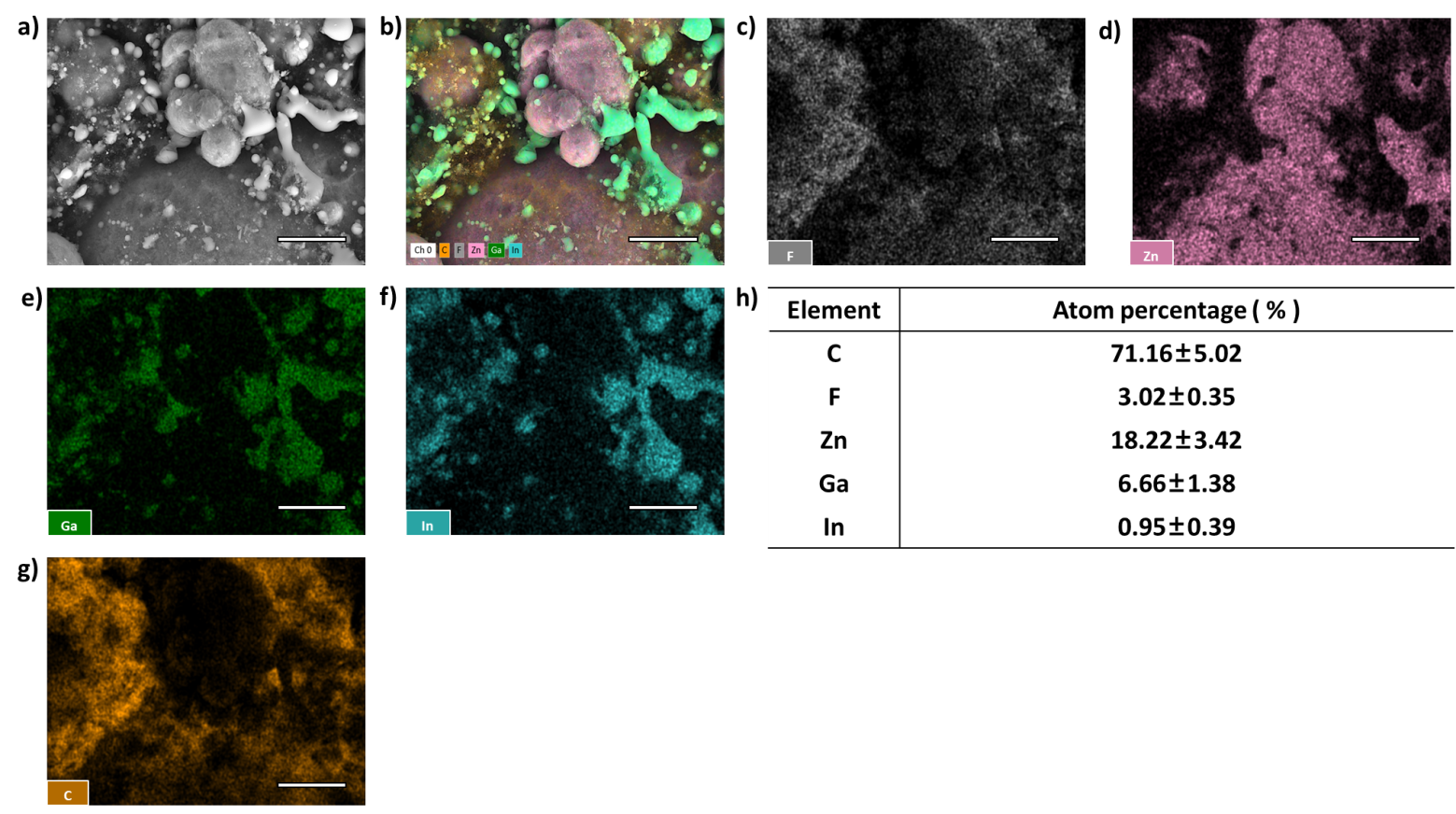


**Figure S4.** High-magnification morphology and elemental characterization of Zn film with LM electrode. (a) SEM image. EDS mapping results of (b) all, (c) F, (d) Zn, (e) Ga, (f) In, (g) C, and the atom percentage table (h) corresponding to b. Scale bar: 5 μm.


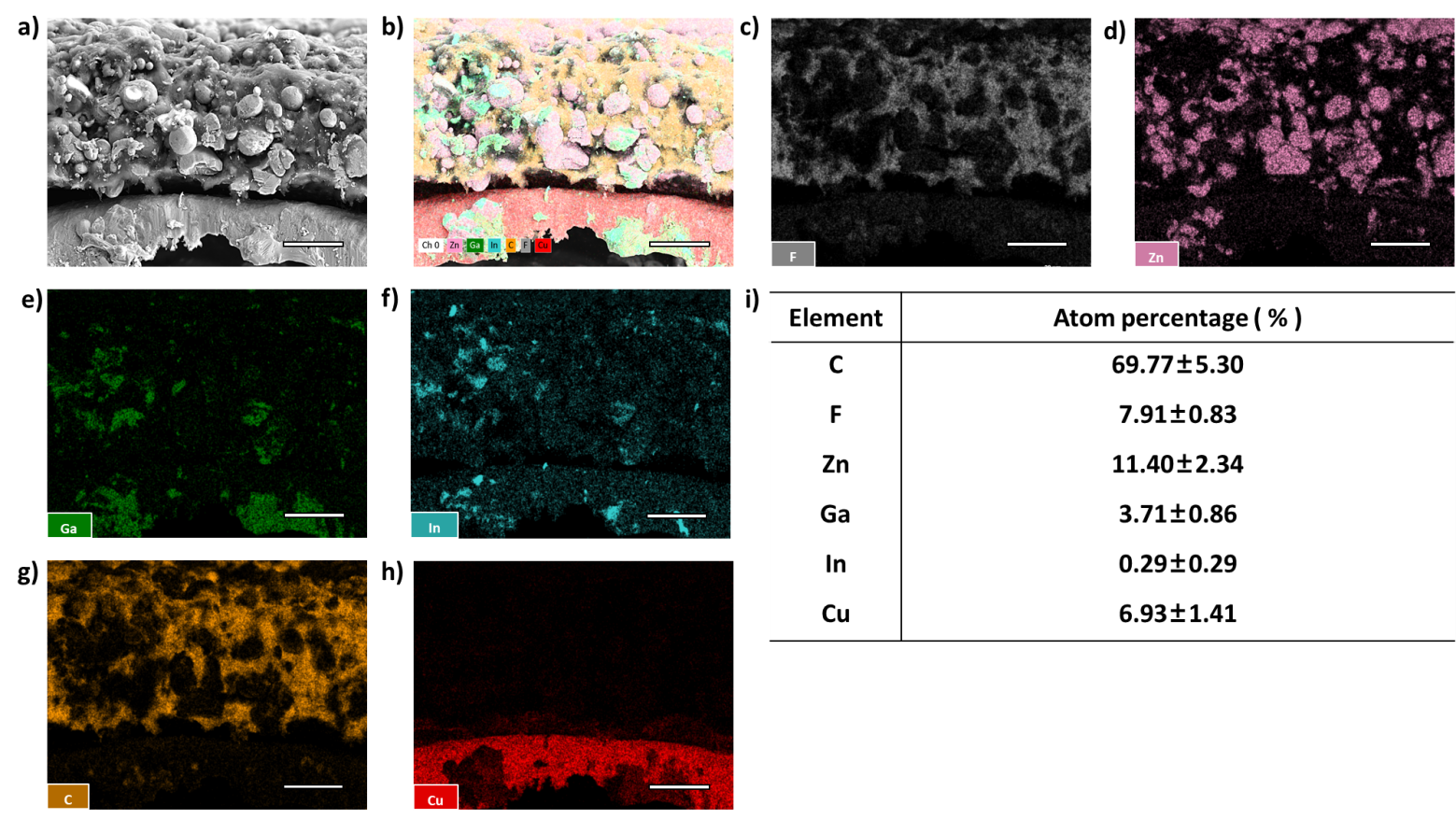


**Figure S5.** Cross-sectional morphology and elemental characterization of Zn film with LM electrode. (a) SEM image. EDS mapping results of (b) all. (c) F, (d) Zn, (e) Ga, (f) In, (g) C, (h) Cu, and the atom percentage table (i) corresponding to b. Scale bar: 20 μm.


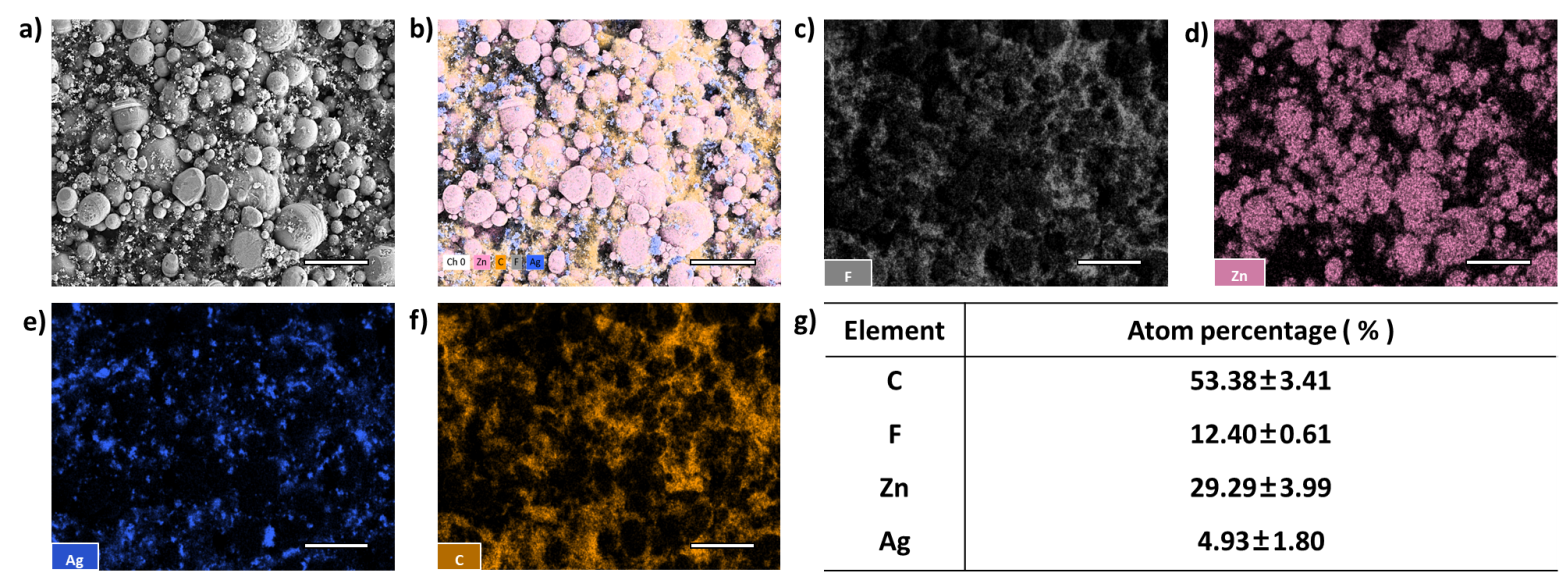


**Figure S6.** Low-magnification morphology and elemental characterization of Zn film with Ag electrode. (a) SEM image. EDS mapping results of (b) all, (c) F, (d) Zn, (e) Ag, (f) C, and the atom percentage table (g) corresponding to b. Scale bar: 20 μm.


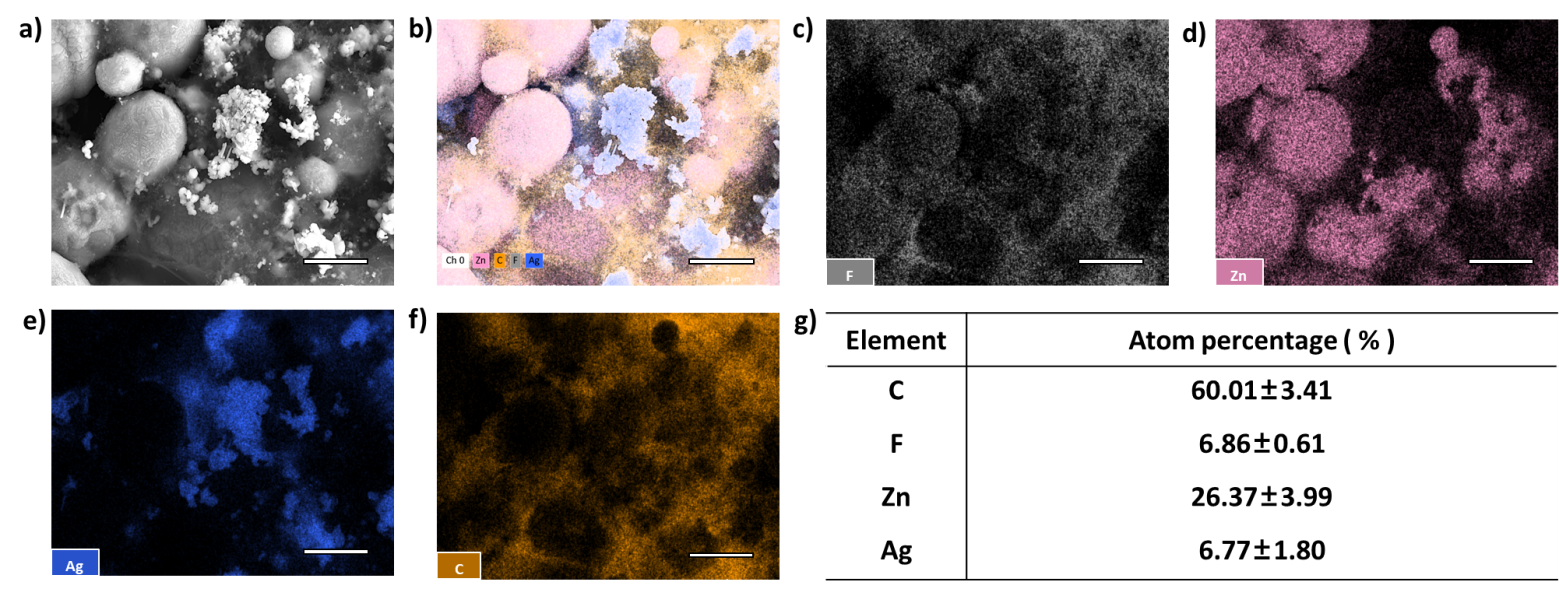


**Figure S7**. High-magnification morphology and elemental characterization of Zn film with Ag electrode. (a) SEM image. EDS mapping results of (b) all, (c) F, (d) Zn, (e) Ag, (f) C, and the atom percentage table (g) corresponding to b. Scale bar: 5 μm.


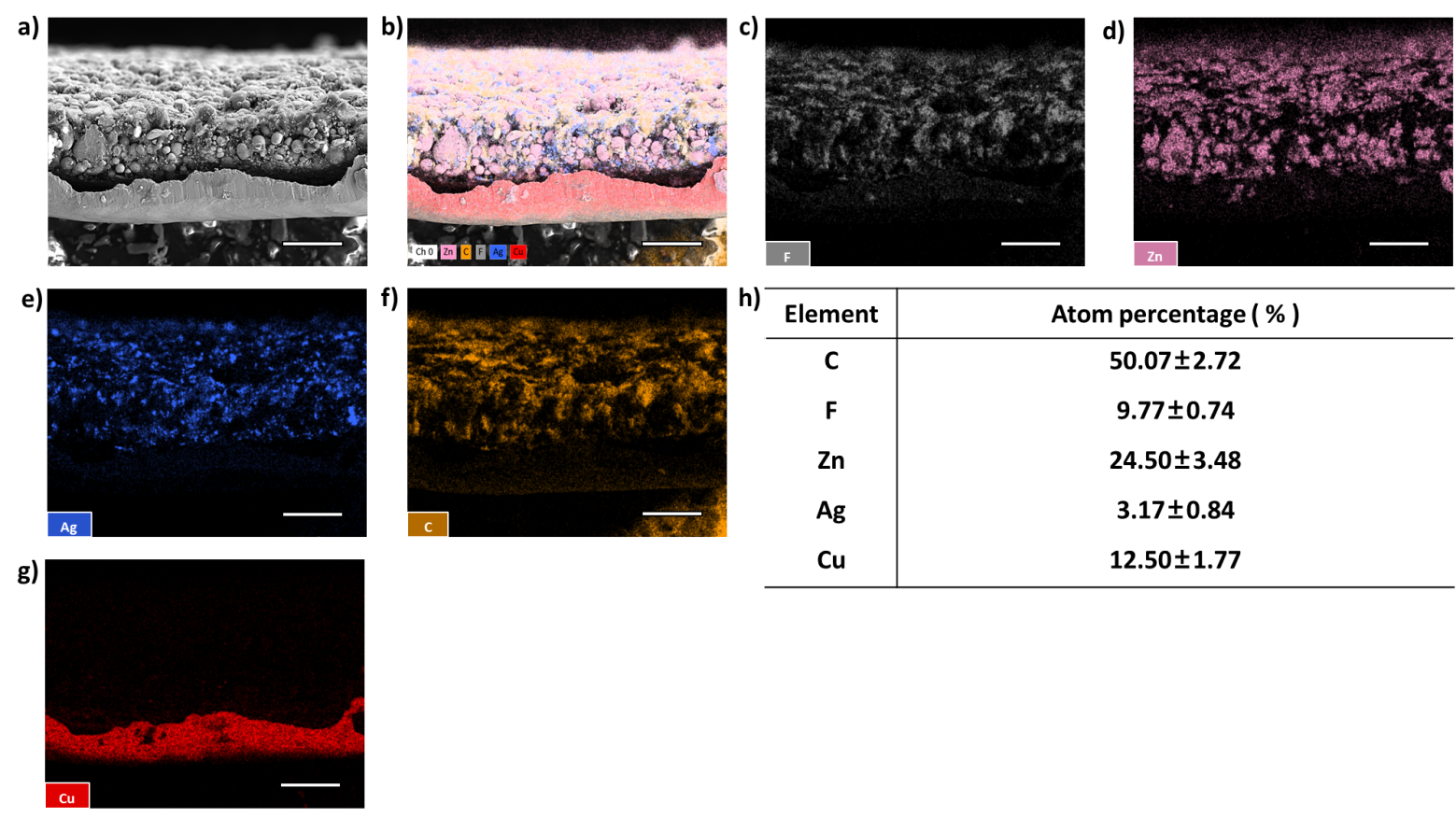


**Figure S8.** Cross-sectional morphology and elemental characterization of the Zn film with Ag electrode. (a) SEM image. EDS mapping results of (b) all, (c) F, (d) Zn, (e) Ag, (f) C, (g) Cu, and the atom percentage table (h) corresponding to b. Scale bar: 20 μm.


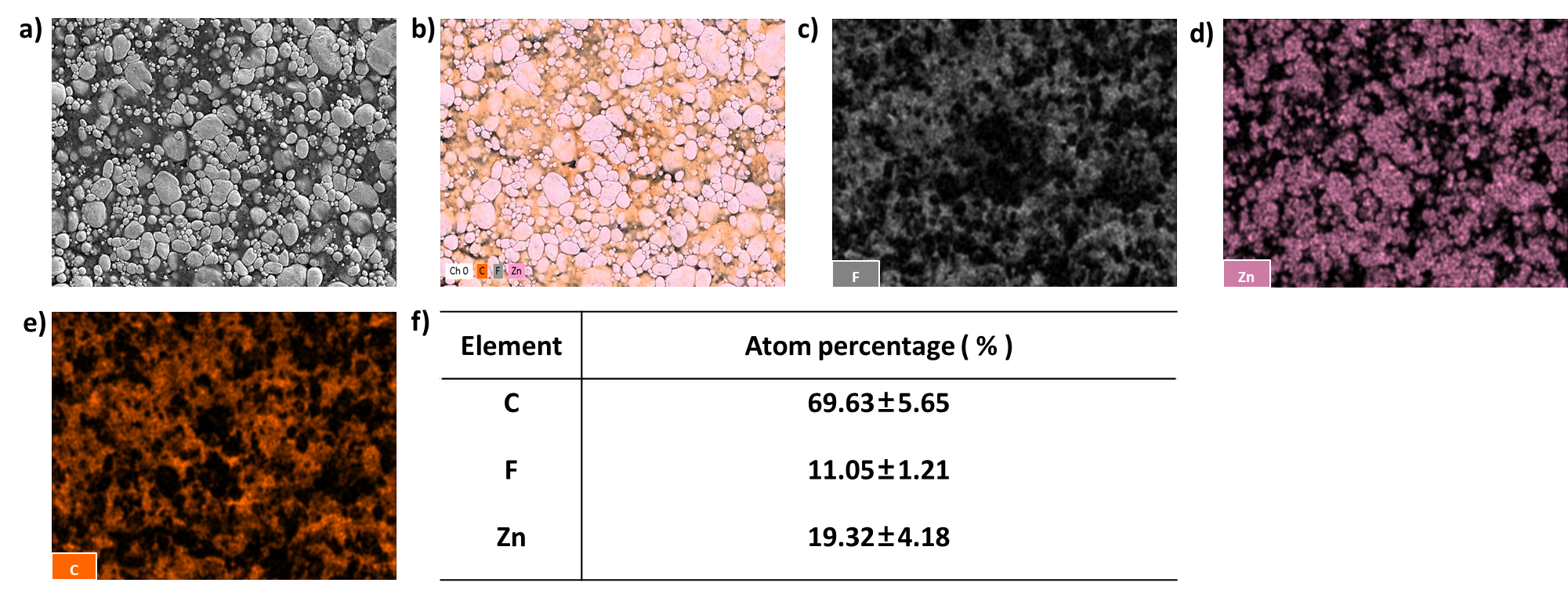


**Figure S9.** Low-magnification morphology and elemental characterization of the bare Zn film electrode. (a) SEM image. EDS mapping results of (b) all, (c) F, (d) Zn, (e) C, and the atom percentage table (f) corresponding to b. Scale bar: 20 μm.


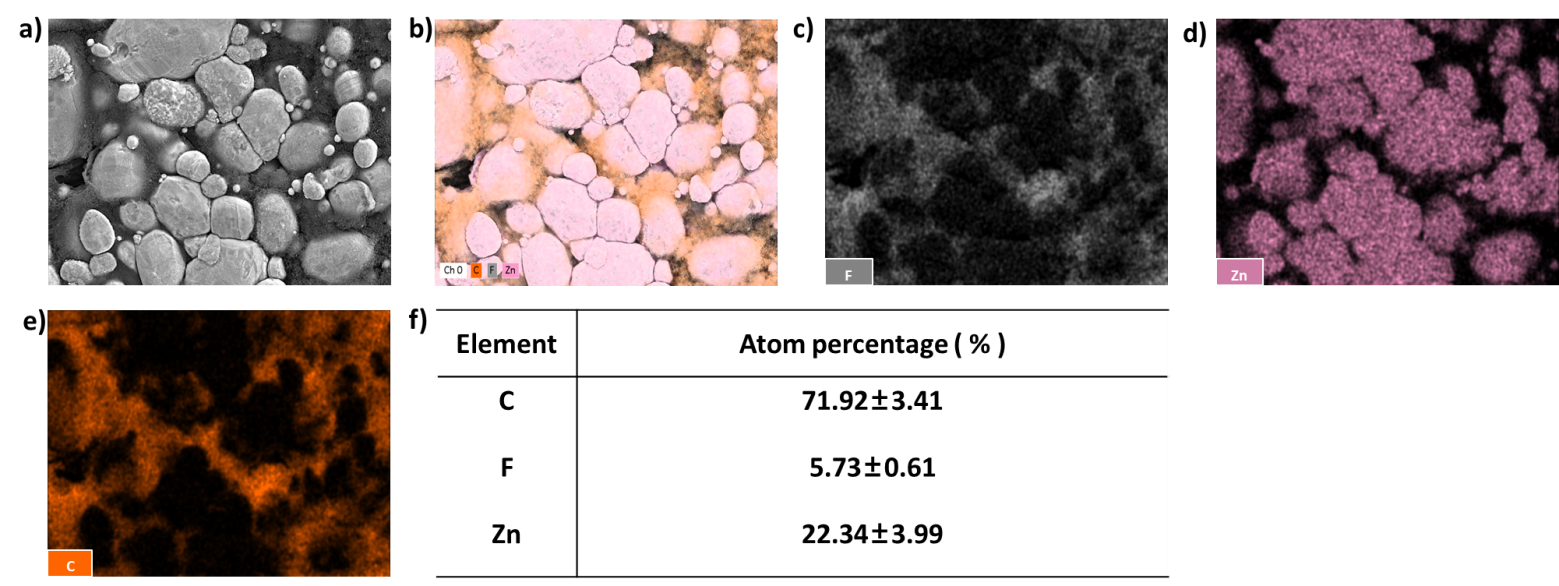


**Figure S10.** High-magnification morphology and elemental characterization of bare Zn film electrode. (a) SEM image. EDS mapping results of (b) all, (c) F, (d) Zn, (e) C, and the atom percentage table (f) corresponding to b. Scale bar: 5 μm.


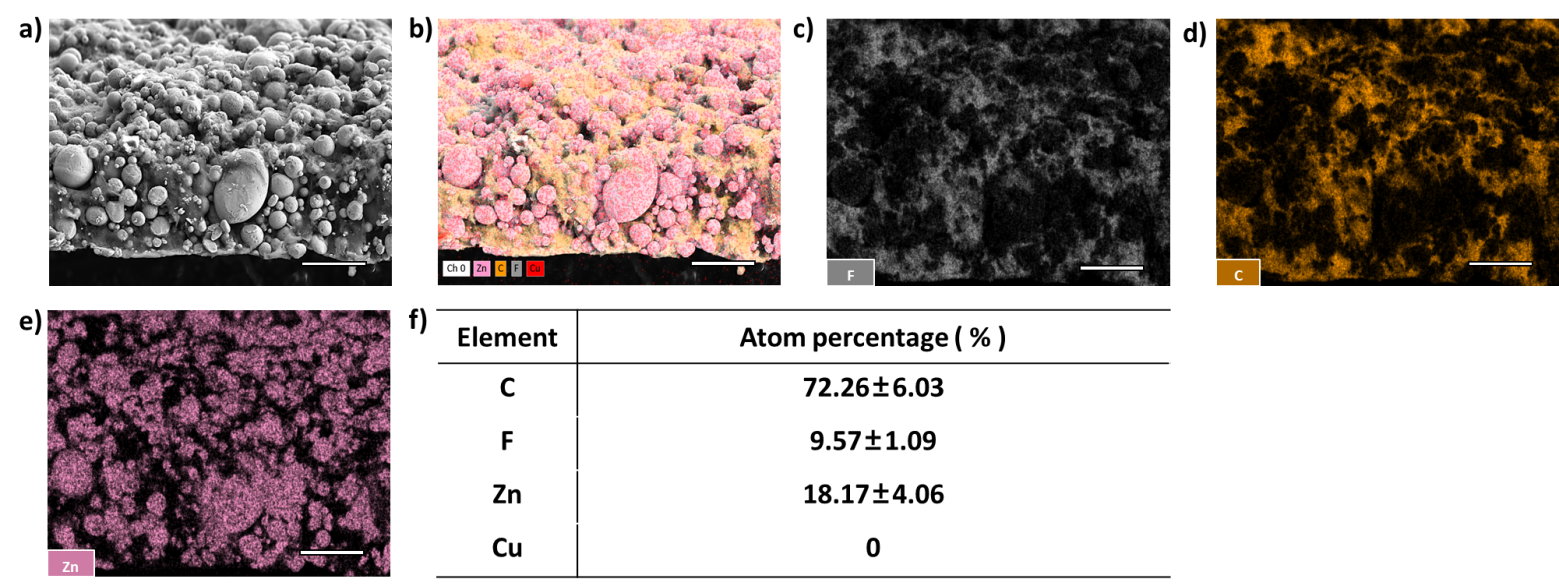


**Figure S11.** Cross-sectional morphology and elemental characterization of the bare Zn film electrode. (a) SEM image. EDS mapping results of (b) all, (c) F, (d) Zn, (e) C, and the atom percentage table (f) corresponding to b. Scale bar: 20 μm. The Cu foil substrate has been removed before the SEM sample preparation.


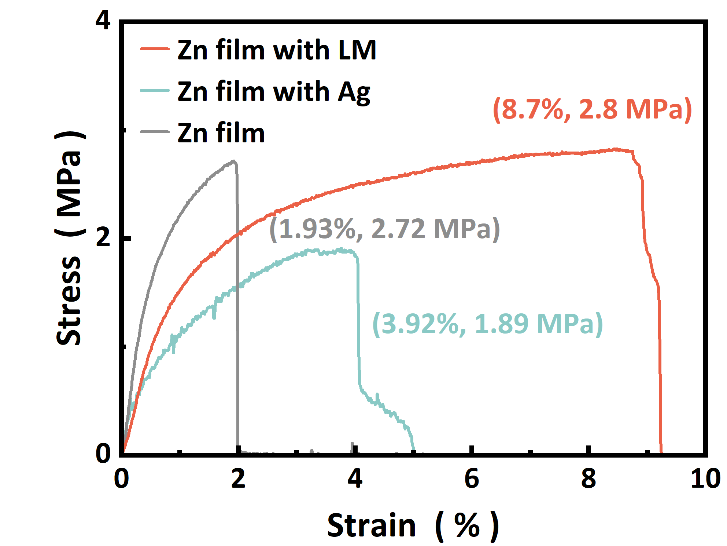


**Figure S12.** Mechanical strength comparison of three different self-standing electrodes.


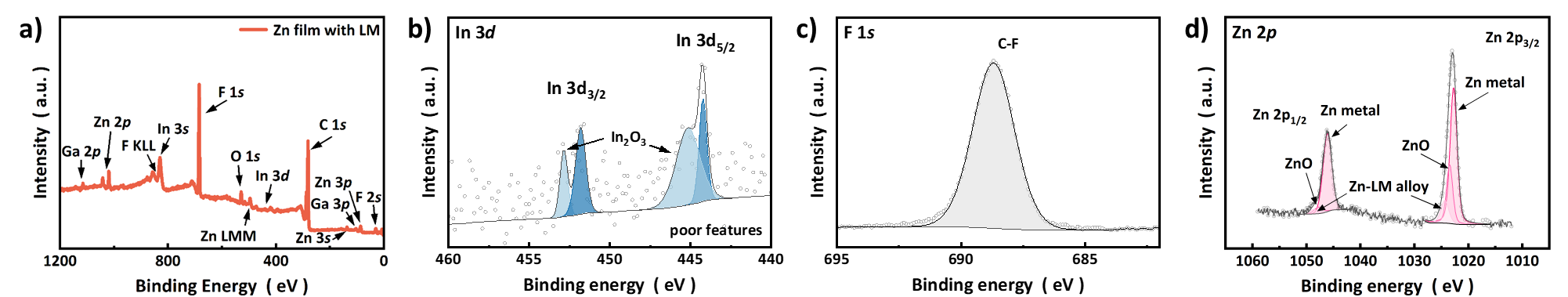


**Figure S13.** Supplementary XPS analysis of the Zn film with LM electrode. (a) Board spectrum, (b) In 3*d*, (c) F 1*s*, and (d) Zn 2*p* spectra.


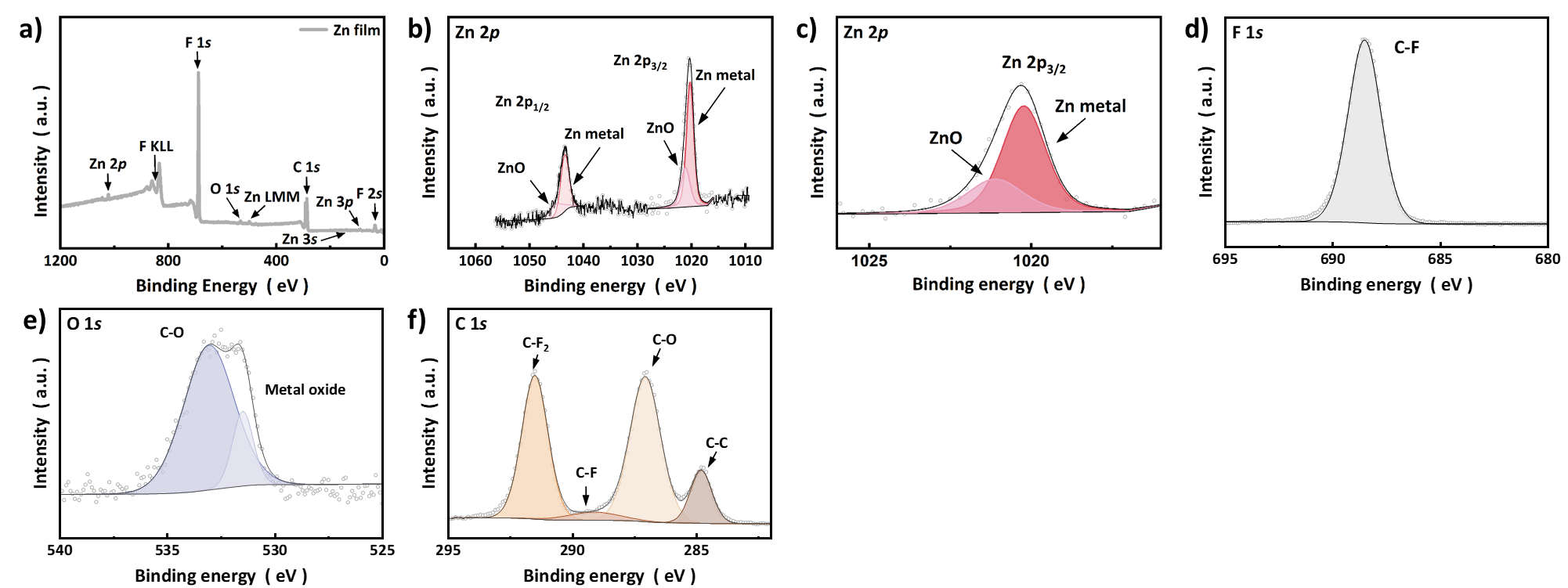


**Figure S14.** Supplementary XPS analysis of the bare Zn film electrode. (a) Board spectrum, (b) Zn 2*p*, (c) Zn 2*p*_3/2_, (d) F 1*s*, (e) O 1*s*, and (f) C 1*s* spectra.


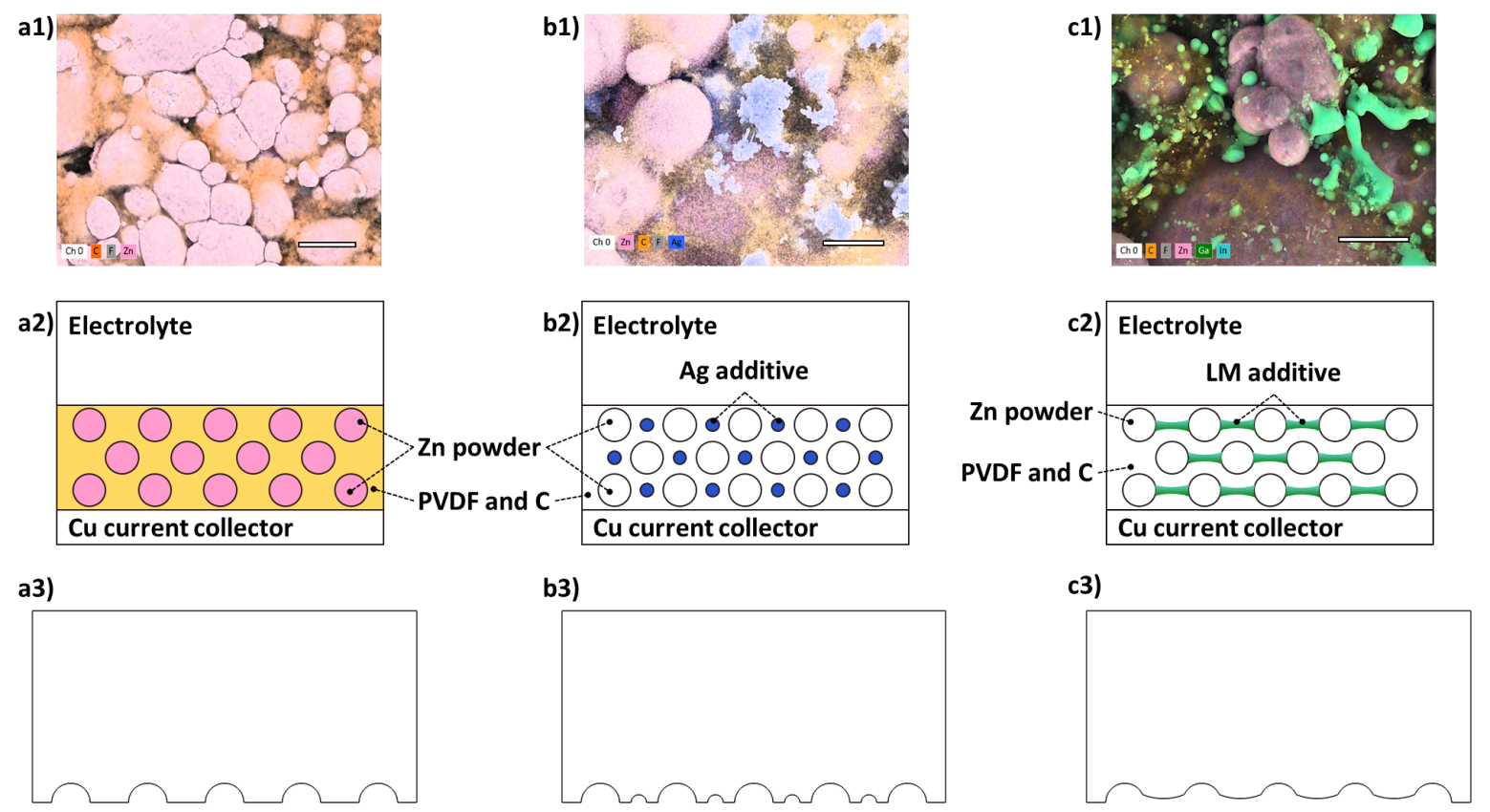


**Figure S15.** Development of simulation models based on EDS results. EDS mappings of (a1) bare Zn film electrode, (b1) Zn film with Ag electrode, and (c1) Zn film with LM electrode. (a2-b2) Derived cross-sectional electrode structures and (a3-c3) simplified top morphology of the electrodes.


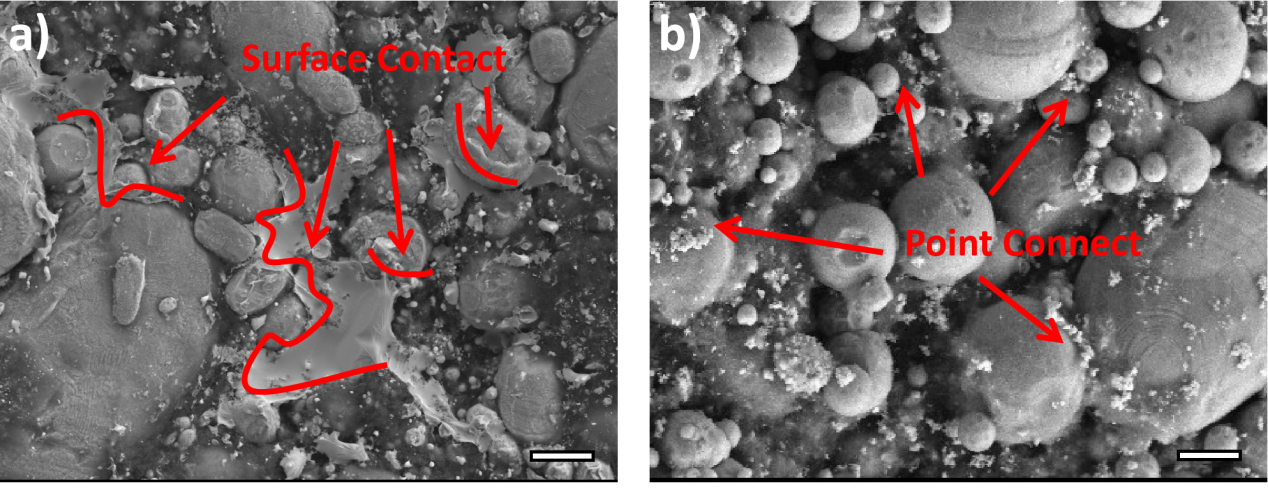


**Figure S16.** Higher-magnification morphologies of the electrodes correspond to Figure 3f. (a) Zn film with LM electrode and (b) Zn film with Ag electrode. Scale bar: 5 µm.


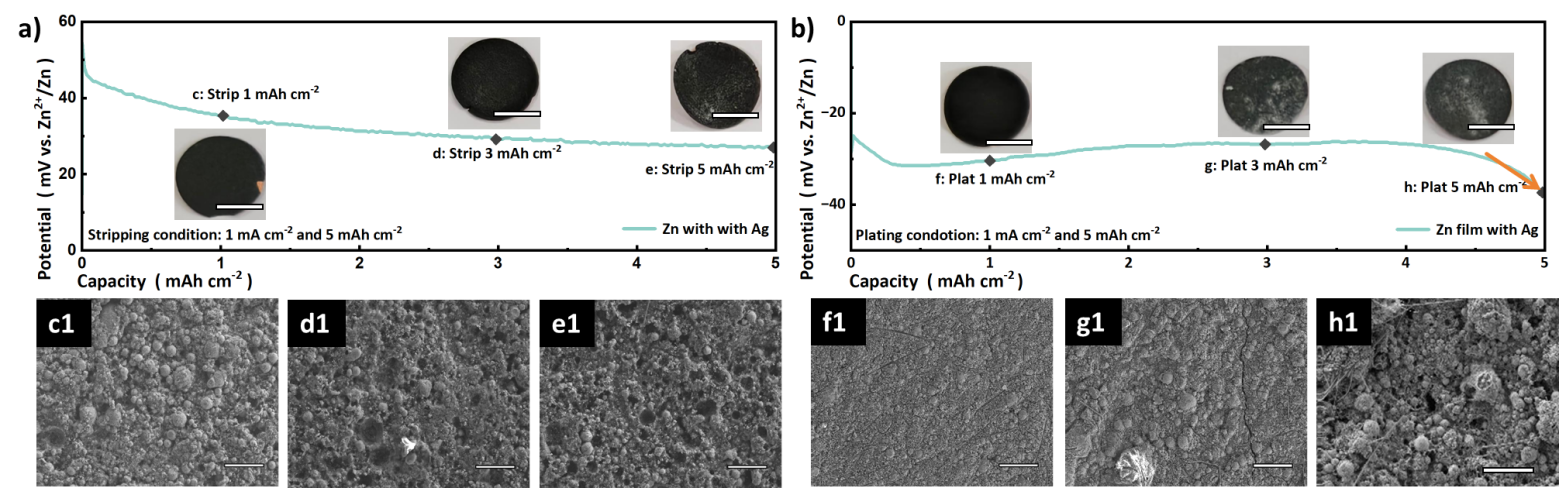


**Figure S17.** Morphological characterization of the Zn film with Ag electrode. Capacity-potential profile of Zn film with Ag electrode undergoes (a) Zn stripping and (b) Zn plating of 5 mAh cm^-2^. The insets show the corresponding optical photograph of stripped/plated electrodes. (c1-h1) SEM images of low magnification at corresponding plating or stripping scenarios to Figure 4 G2-L2. Scale bar: 0.5 cm for (a and b), and 20 µm for (c1–h1).


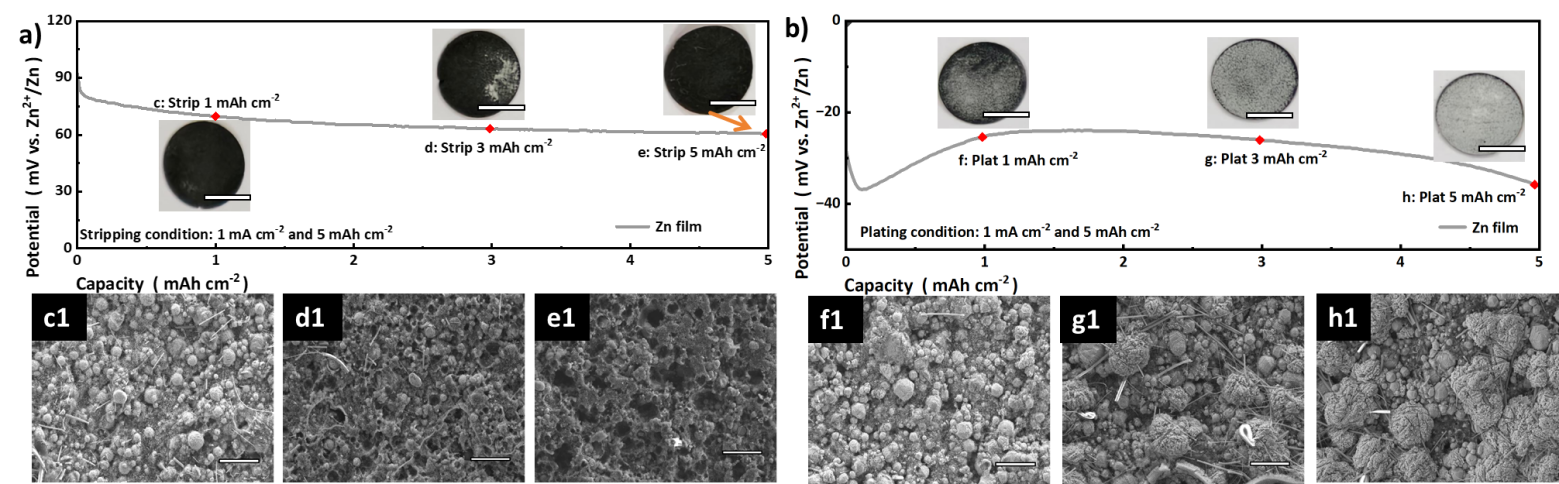


**Figure S18.** Morphological characterization of the bare Zn film electrode. Capacity-potential profile of bare Zn film electrode undergoes (a) Zn stripping and (b) Zn plating of 5 mAh cm^-2^. The insets show the corresponding optical photograph of stripped/plated electrodes. (c1-h1) SEM images of low magnification at corresponding plating or stripping scenarios to Figure 4 G3-L3. Scale bar: 0.5 cm for (a and b), and 20 µm for (c1–h1).


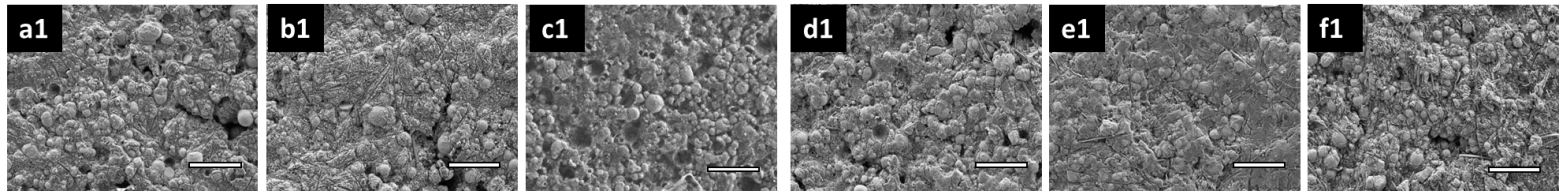


**Figure S19.** Morphological characterization of the Zn film with LM electrode. (a1-f1) SEM images of low magnification at different plating or stripping scenarios corresponding to Figure 4 G1-L1. Scale bar: 20 µm for (a1–f1).


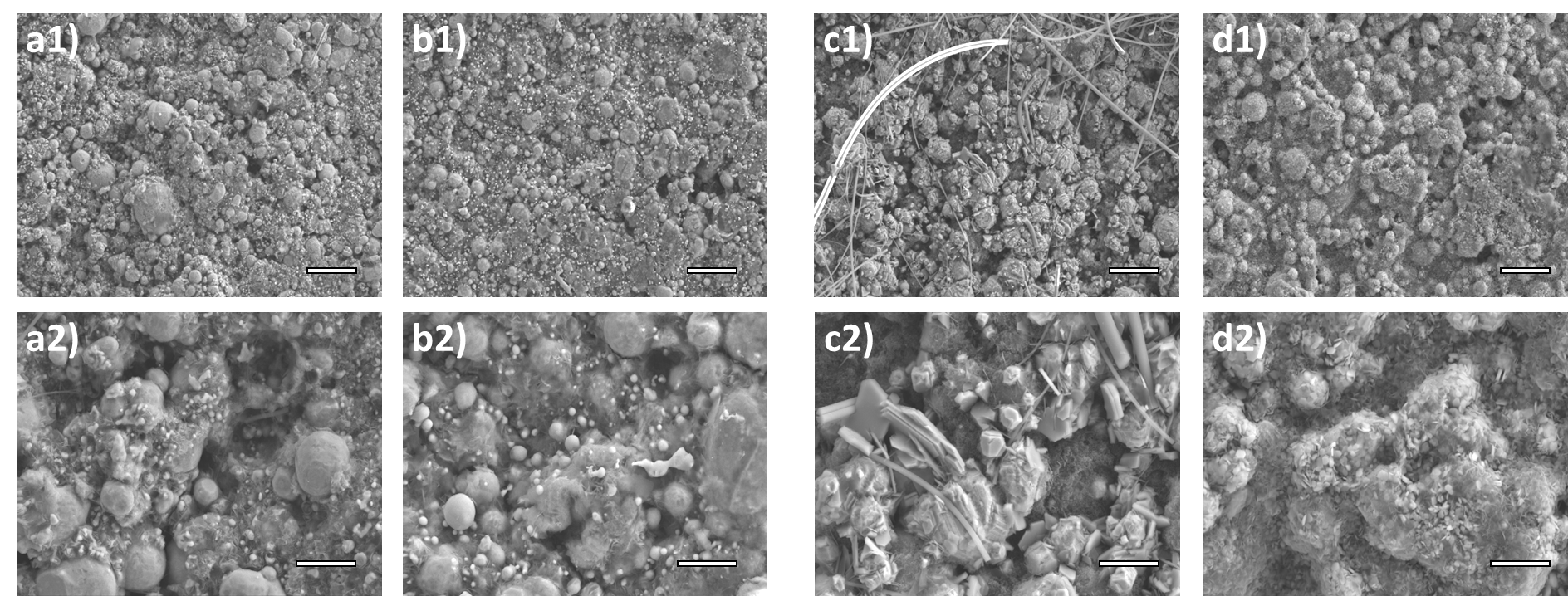


**Figure S20.** Morphological characterization of the Zn film with LM and Ag electrode after plating/stripping under the condition of 10 mA cm^-2^ and 3 mAh cm^-2^. Zn (a1-a2) plating and (b1-b2) stripping morphology of the Zn film with LM electrode. Zn (c1-c2) plating and (d1-d2) stripping morphology of Zn film with Ag electrode. cale bars: 20 μm for (a1-d1), and 5 μm for (a2-d2).


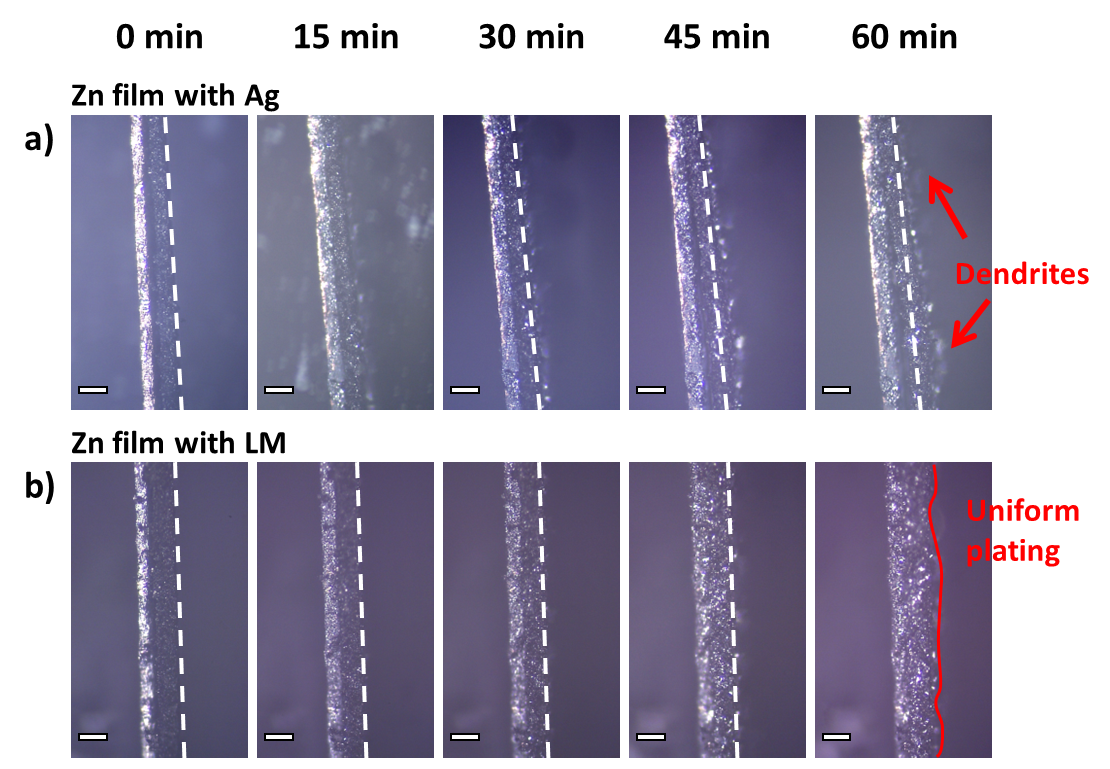


**Figure S21.** *In-situ* optical microscopy characterization of two different electrodes. Cross-sectional morphologies of (a) Zn film with Ag electrode and (b) Zn film with LM electrode. Plating current density: 25 mA cm^-2^. Scale bar: 50 µm.


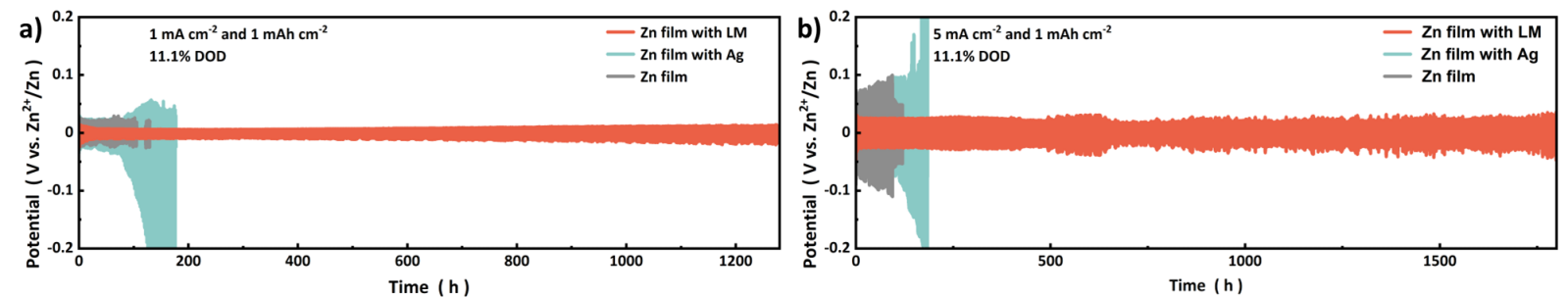


**Figure S22.** Voltage profiles of Zn||Zn symmetrical cells with the cycling conditions of (a) 11.1% DOD at 1 mA cm^-2^/1 mAh cm^-2^ and (b) 11% DOD at 5 mA cm^-2^/1 mAh cm^-2^.


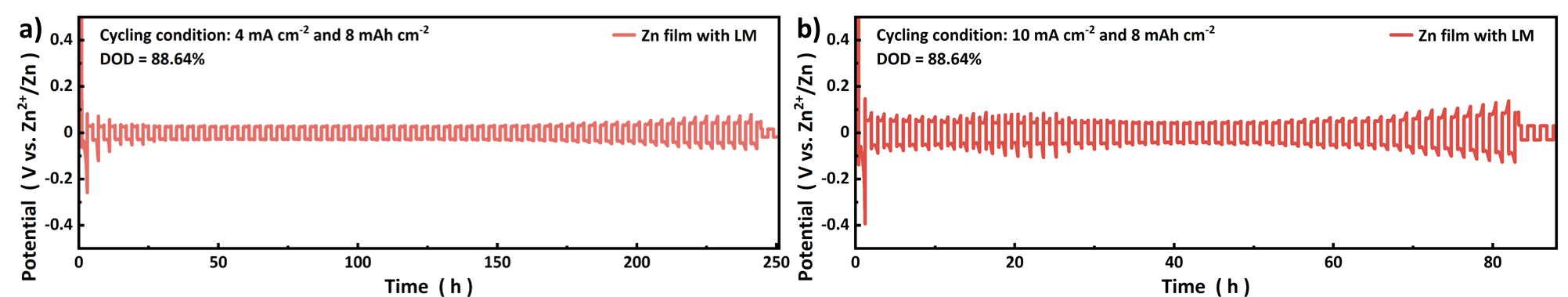


**Figure S23.** Voltage profiles of Zn||Zn symmetrical cells with the cycling conditions of (a) 88.64% DOD at 4 mA cm^-2^/8 mAh cm^-2^ and (b) 88.64% DOD at 10 mA cm^-2^/8 mAh cm^-2^.


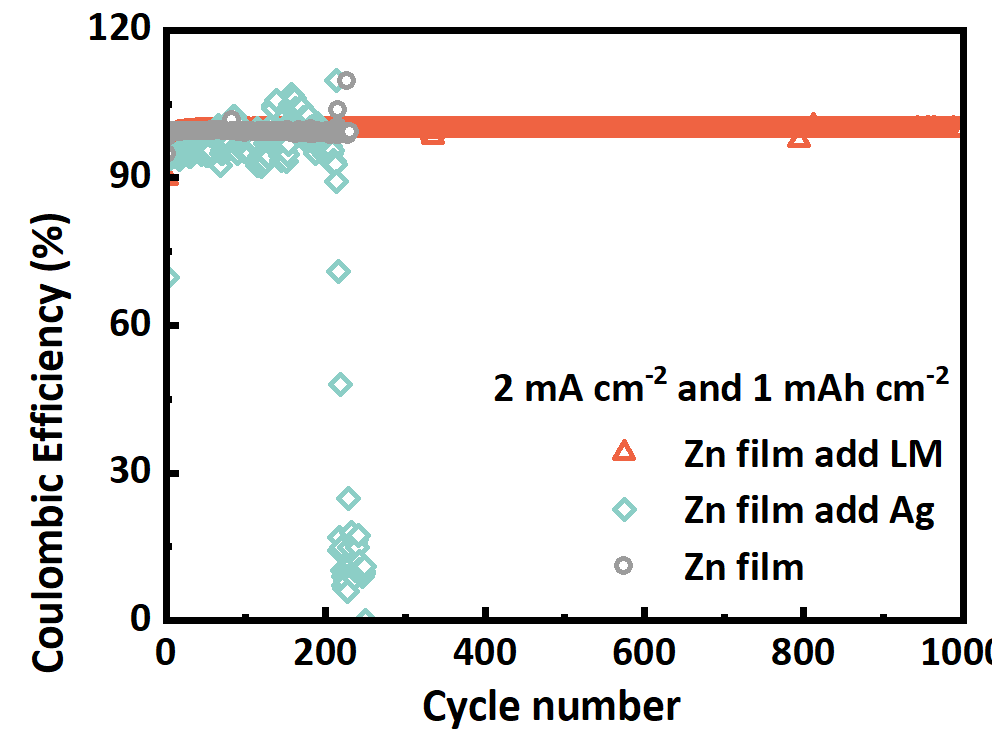


**Figure S24.** CE profile for the half-cells cycling at 2 mA cm^-2^ and 1 mAh cm^-2^.


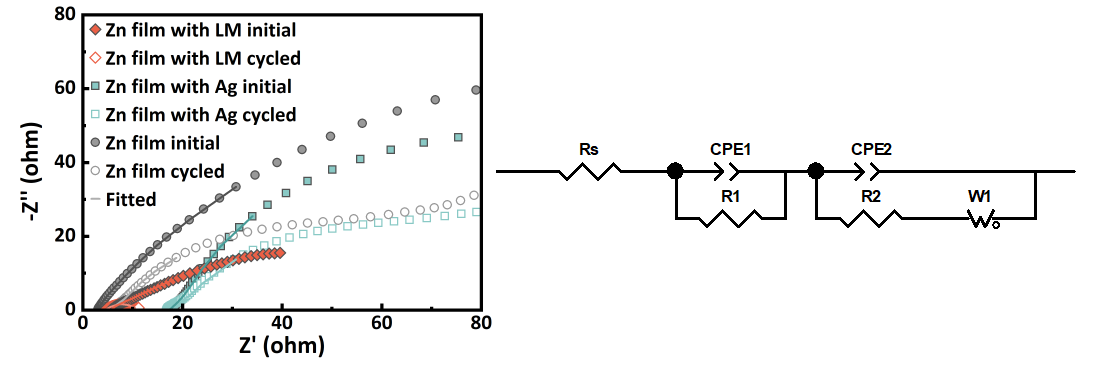


**Figure S25.** EIS plots with a broad region and an equivalent circuit diagram corresponding to Figure 5e.


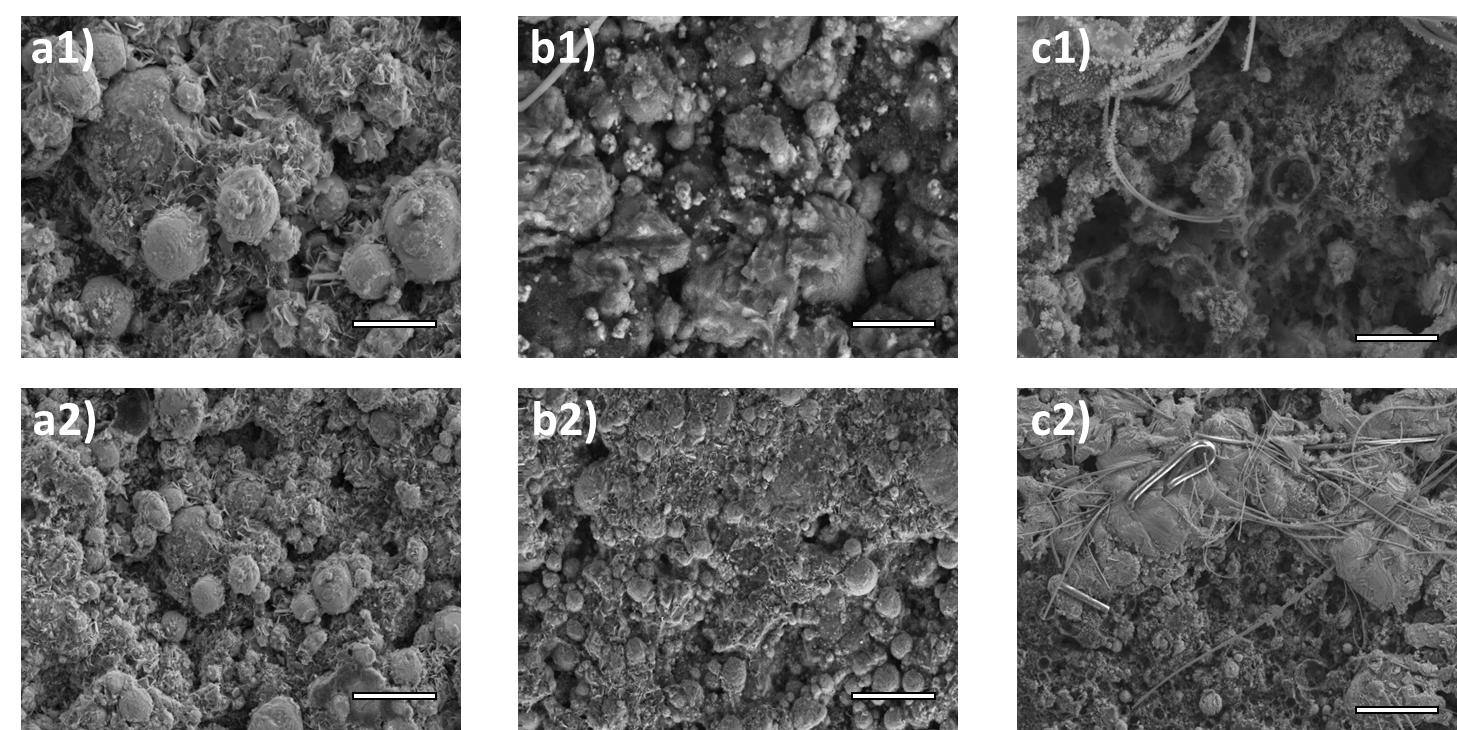


**Figure S26.** Electrode morphology characterization after 100 cycles at 10 mA cm^-2^ in symmetric cells. (a1-a2) Zn film with LM electrode, (b1-b2) Zn film with Ag electrode, and (c1-c2) Zn film electrode. Scale bars: 5 μm for (a1-c1), and 20 μm for (a2-c2).


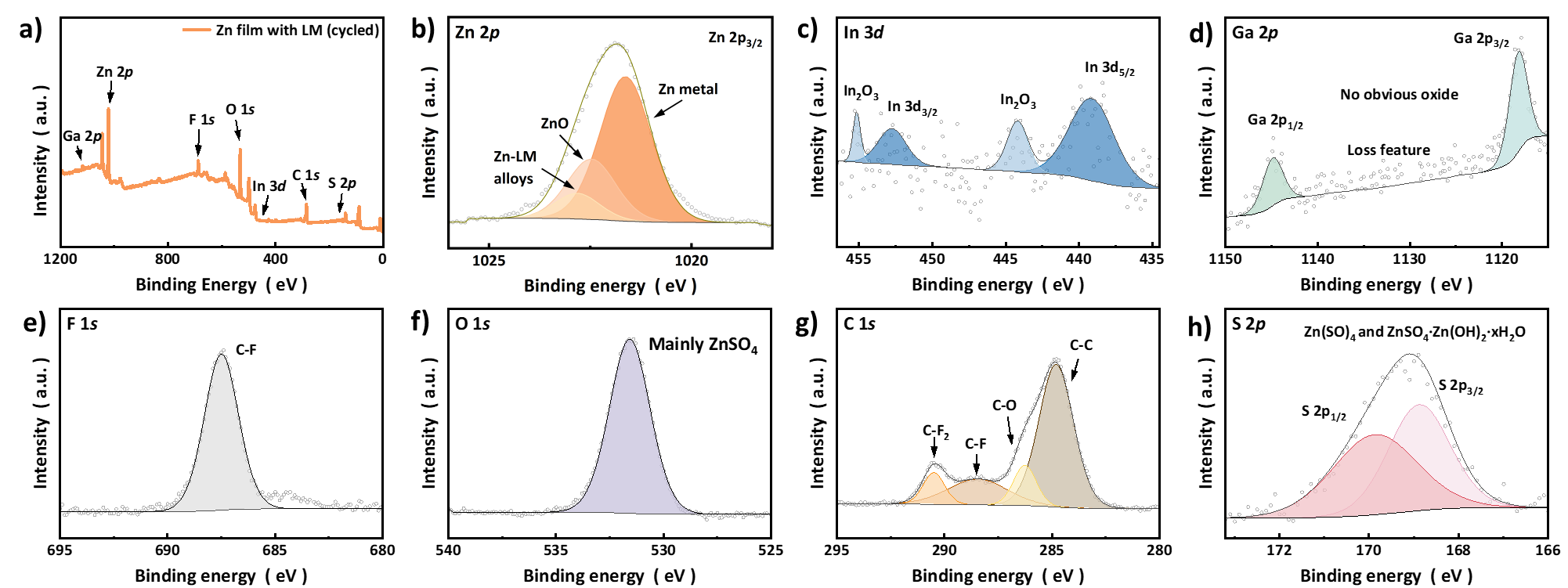


**Figure S27.** XPS analysis of the cycled Zn film with LM electrode. (a) Board spectrum, (b) Zn 2p, (c) In 3d, (d) Ga 2p, (e) F 1s, (f) O 1s, (g) C 1s, and (h) S 2p spectra.


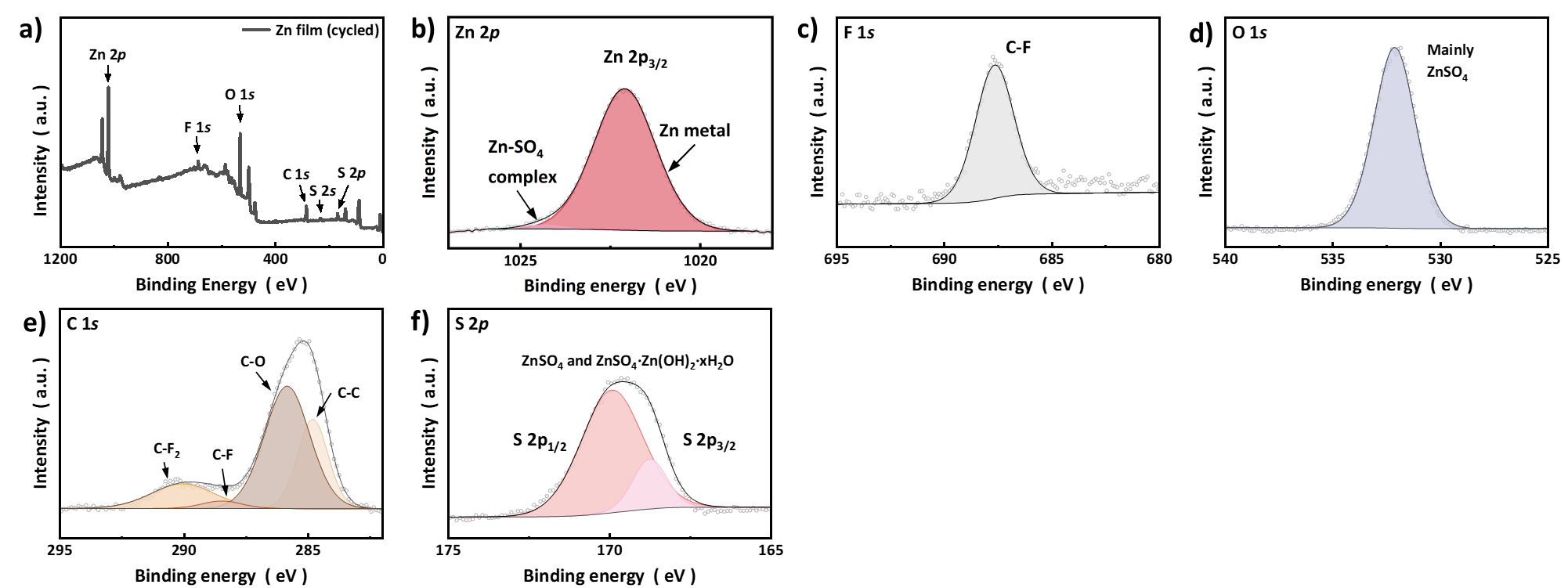


**Figure S28.** XPS analysis of the cycled bare Zn film electrode. (a) Board spectrum, (b) Zn 2p, (c) F 1s, (d) O 1s, (e) C 1s, and (f) S 2p spectra.


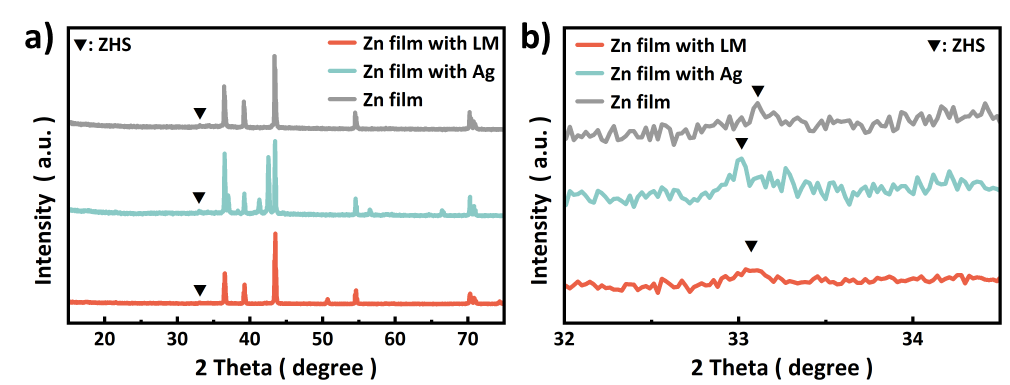


**Figure S29.** XRD patterns of the cycled three electrodes. (a) Board spectrum and (b) magnified view.


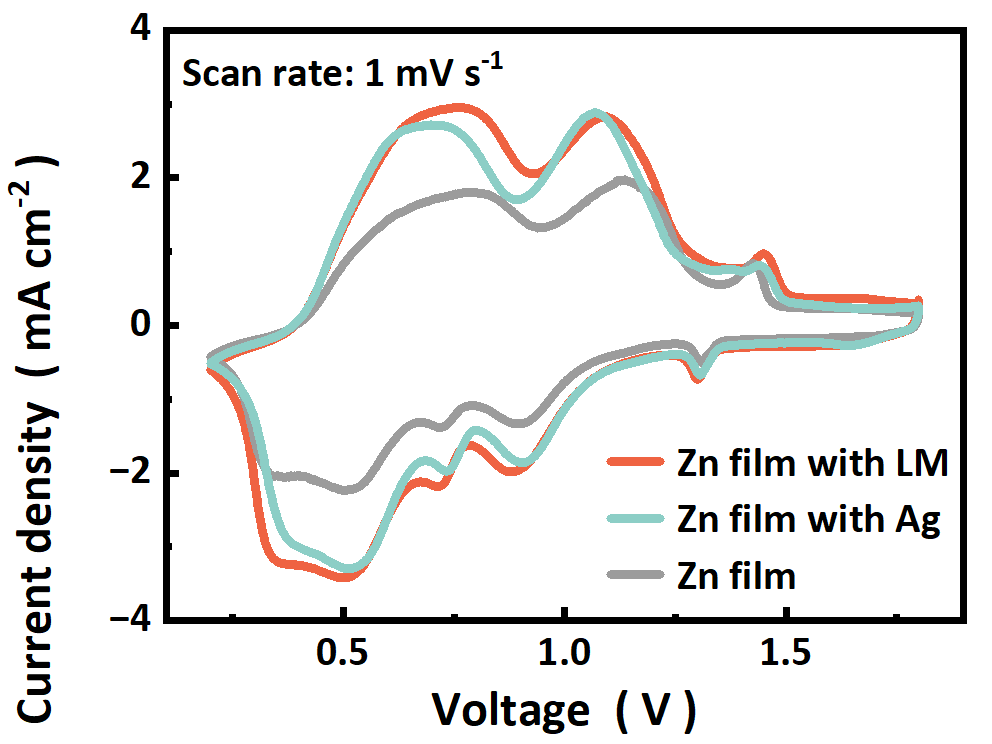


**Figure S30.** CV curves of assembled full cells using three different ZP film electrodes.


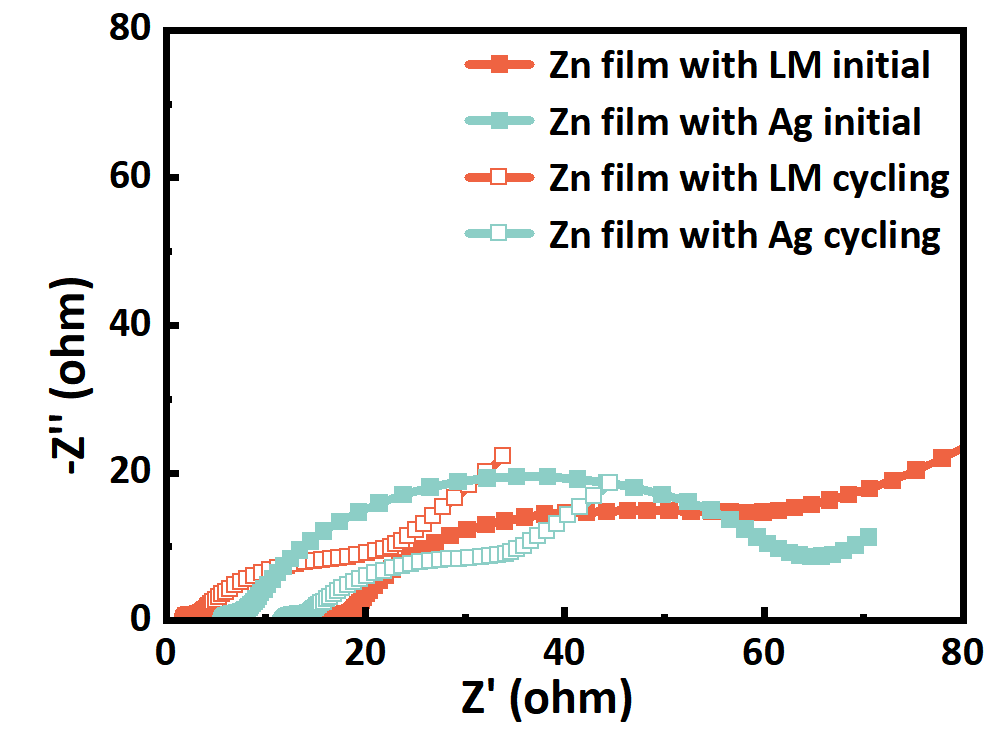


**Figure S31.** EIS plots of assembled full cells (NVO||Zn film with LM or Ag) before and after 10 cycles at 10 A g^-1^.


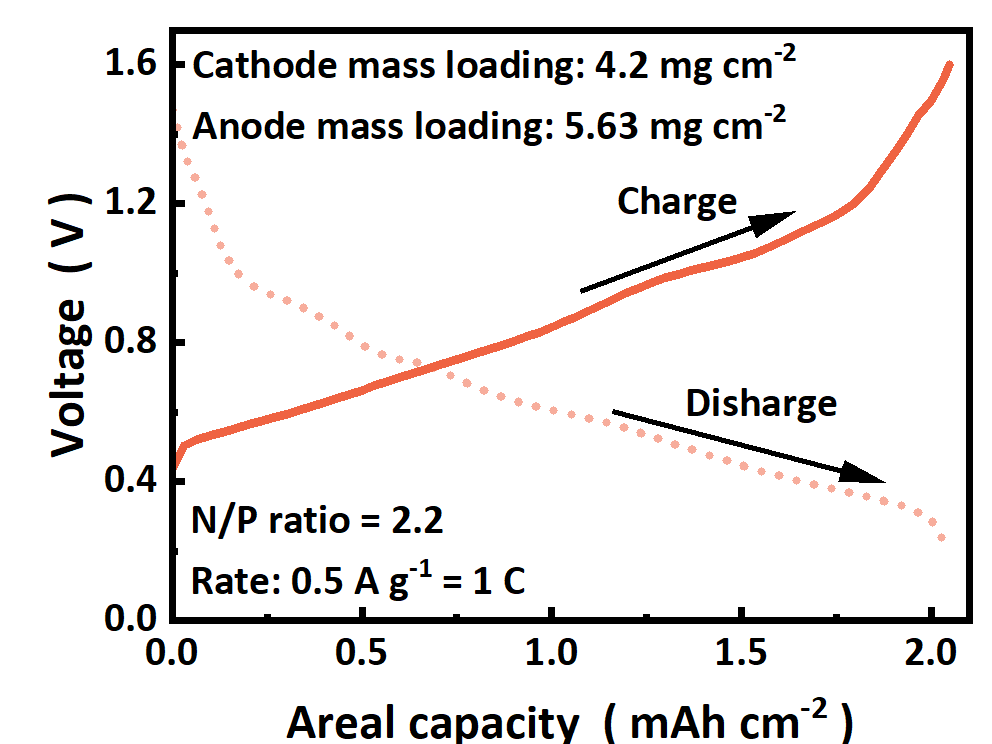


**Figure S32.** Prepared NVO||Zn film with LM full cell with higher cathode mass loading and lower N/P ratio.


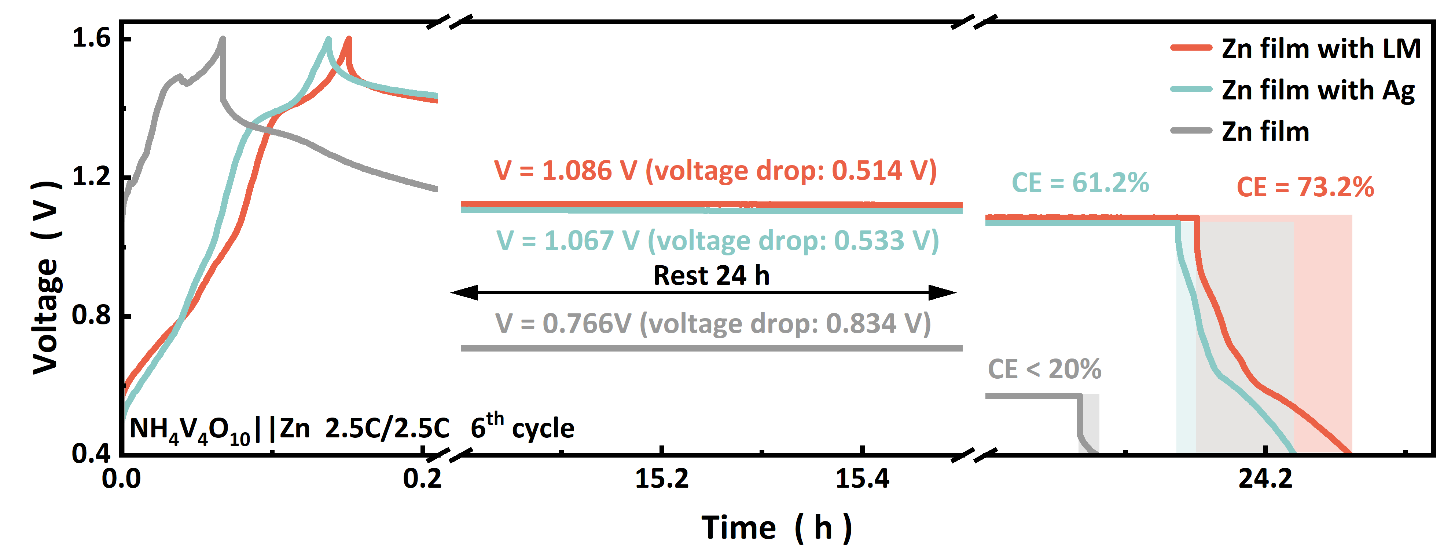


**Figure S33.** Self-discharge performance comparison of three different full cells.


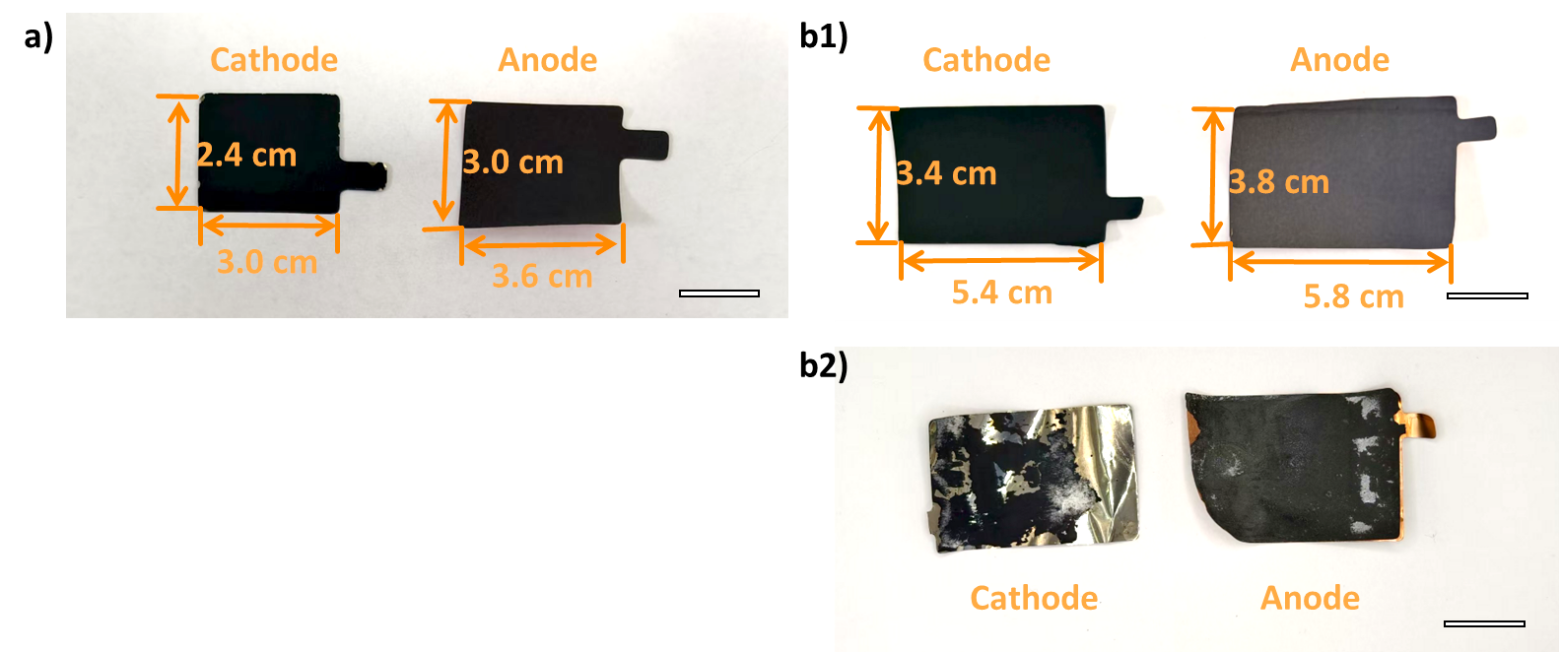


**Figure S34.** Prepared electrodes for pouch cells with different sizes. (a) Smaller one and (b1) larger one. (b2) Optical photograph of the cycled larger electrodes. Scale bars: 2 cm.


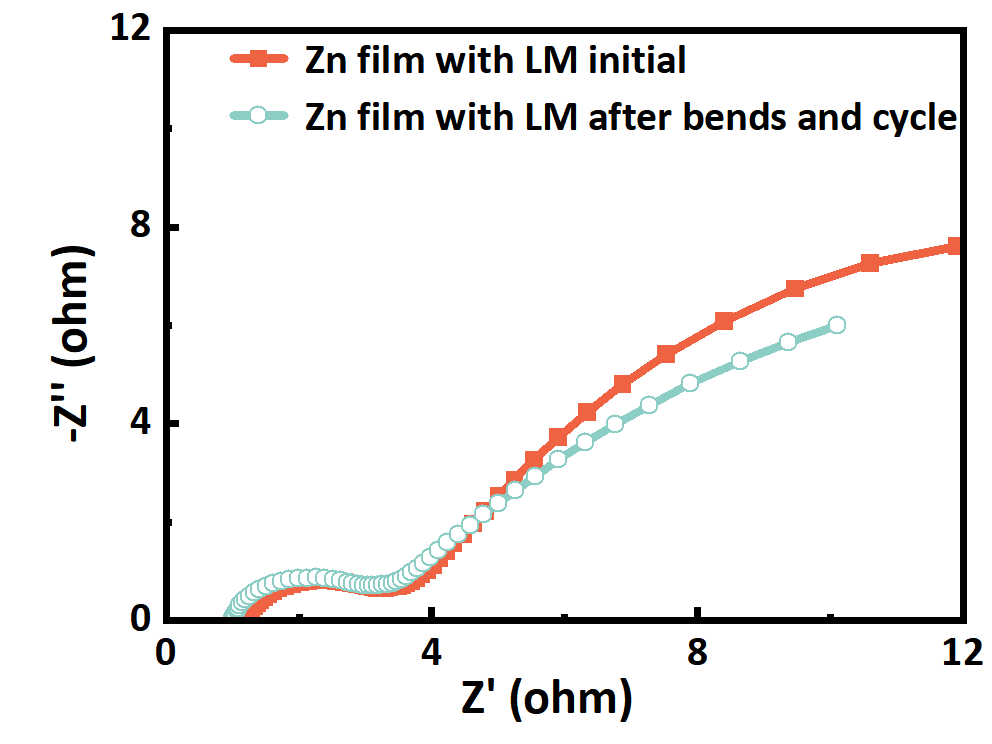


**Figure S35.** EIS comparison of the pouch full cell before and after cycling and bending.

**
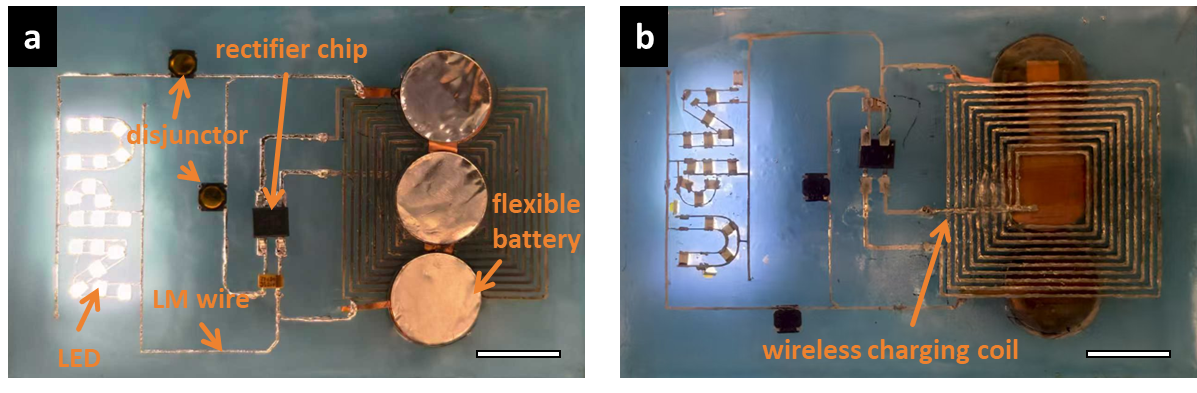
**

**Figure S36.** Detailed components of the integrated flexible device. (a) Front-side and (b) back-side. Scale bars: 1 cm.


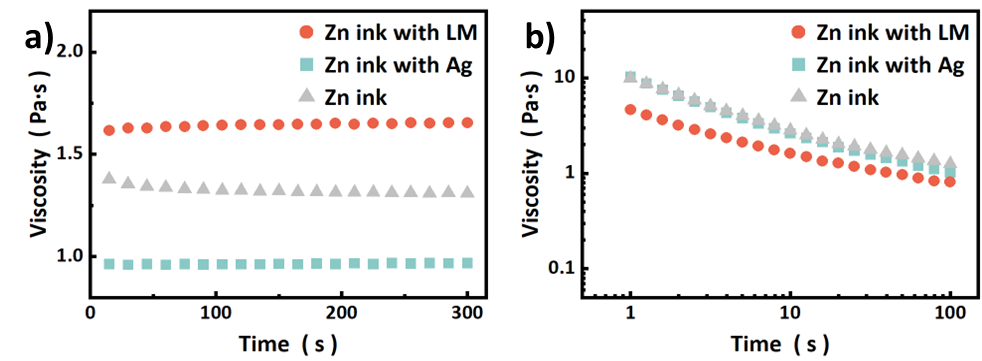


**Figure S37**. Rheological properties of the three different slurries. (a) Static stability and (b) shear-thinning behavior.


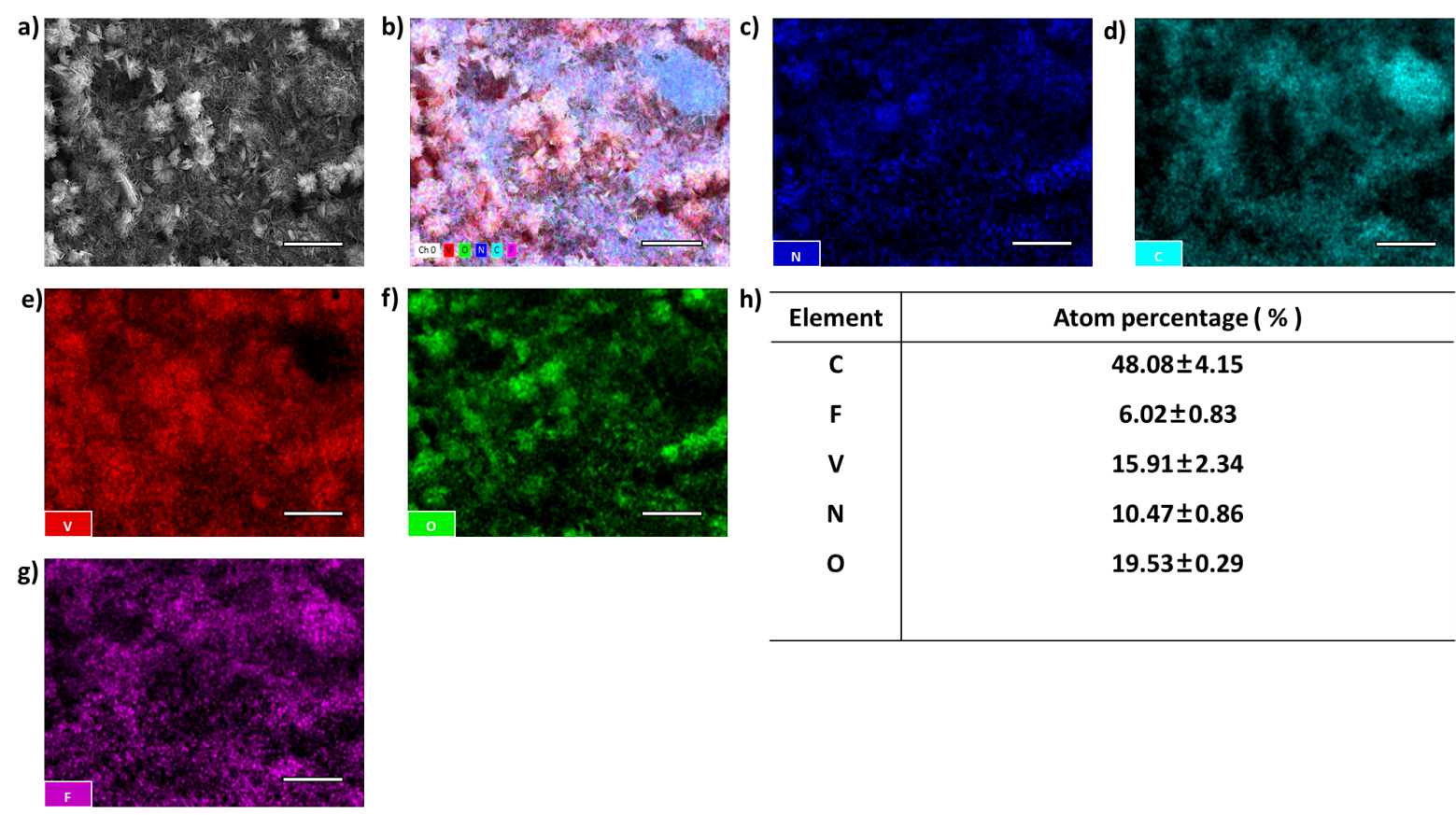


**Figure S38.** Morphological and elemental characterization of NH_4_V_4_O_10_ cathode. (a) SEM image. EDS mappings of (b) all, (c) N, (d) C, (e) V, (f) O, (g) F, and the atom percentage table (h) corresponding to b. Scale bar: 20 μm.

**Table S1.** Symmetric-cell cycling testing details and performance comparison with different Zn powder electrodes.


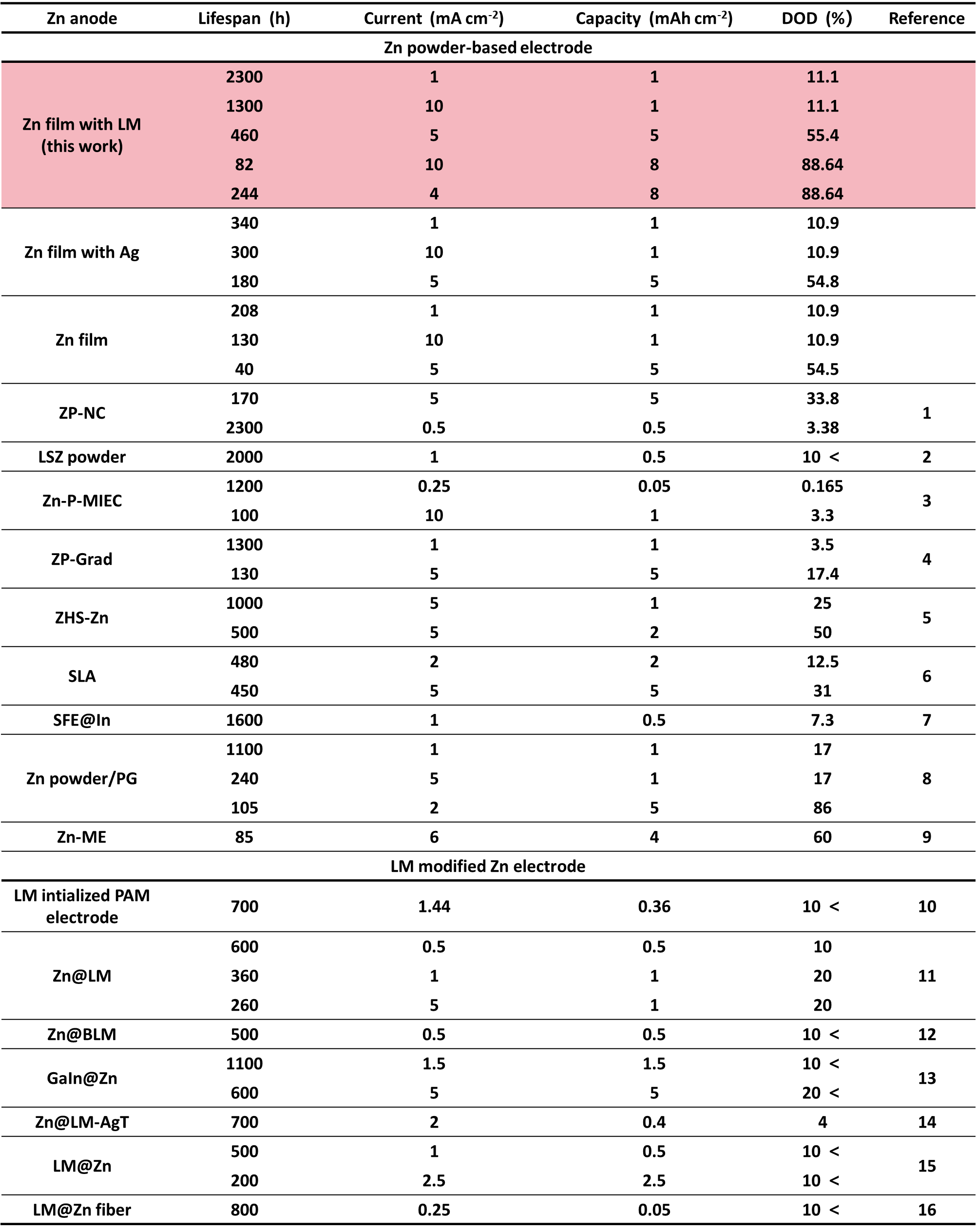


**Table S2.** Full-cell cycling testing details and performance comparison with different Zn powder electrodes in terms of long-term cycling result and power density/energy density calculation.


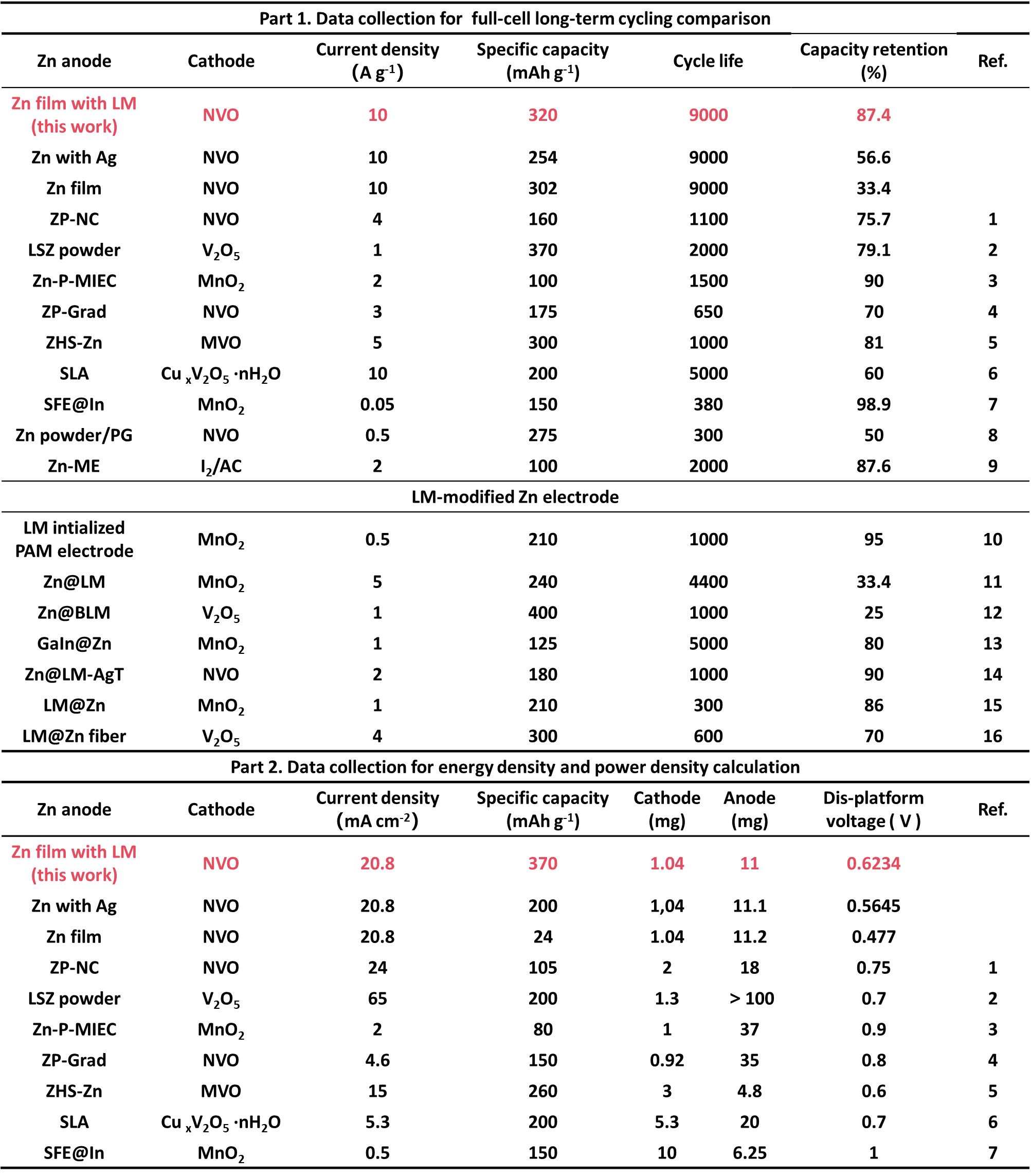


**Table S3.** Simple symmetric-cell cycling performance comparison with different Zn powder electrodes based on the used conductive additives and the corresponding material cost.


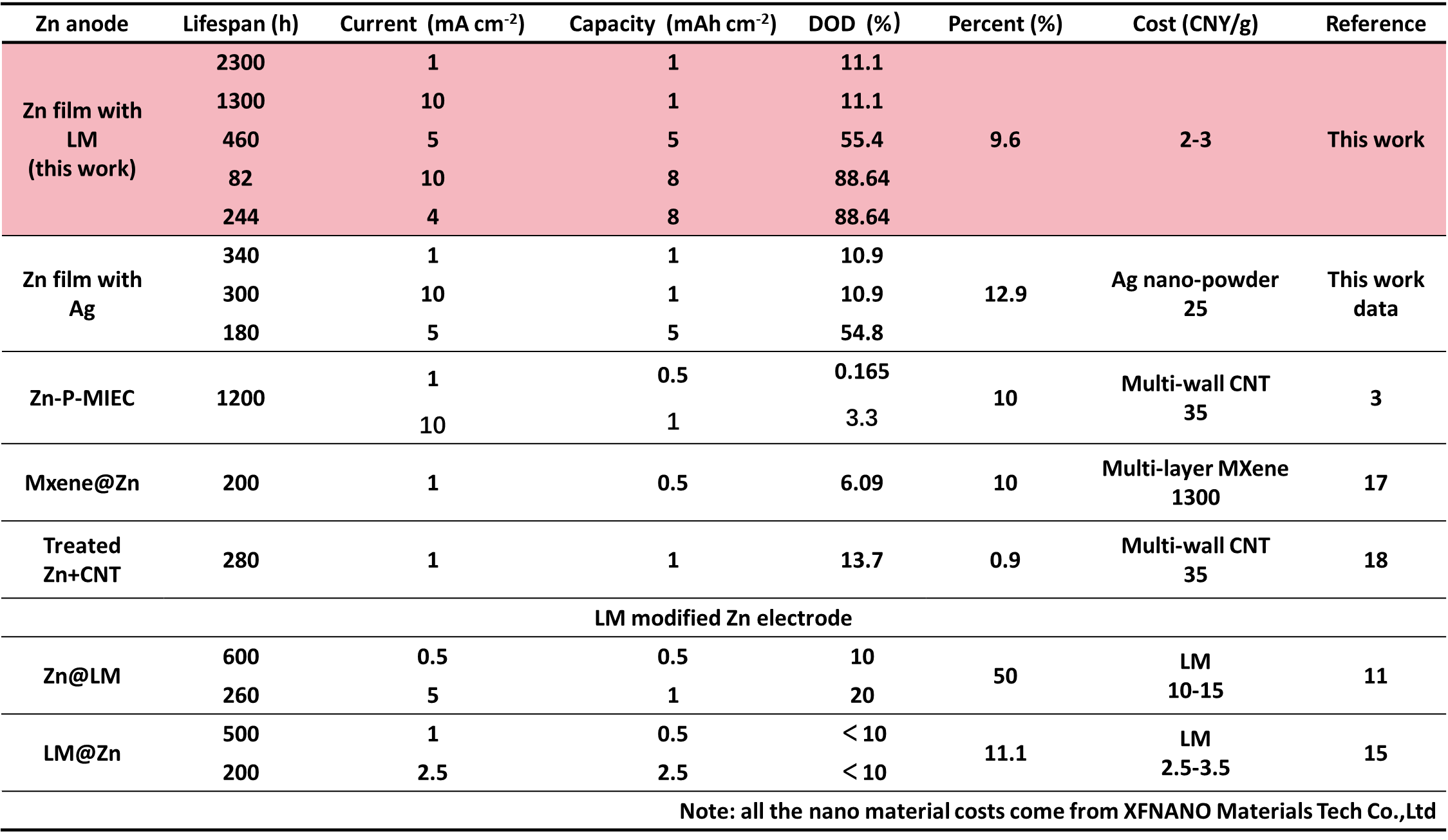


**References**

1. Jin, Y., Jin, K., Ji, W., Zhang, Z., Gan, L., Han, P., Yuan, X., Fu, L., and Wu, Y. (2025). Fabrication of a Robust Zinc Powder Anode via Facile Integration of Copper Nanopowder as a Functional Conductive Medium. Advanced Functional Materials *35*, 2418503. <https://doi.org/10.1002/adfm.202418503>.

2. Kang, H., Kim, S.-H., Ahn, D.B., Wang, X., Wu, Z.-S., and Lee, S.-Y. (2024). Liquid Metal-Skinned Zn Powder Anodes Enabled by Capillary Suspension. ACS Energy Letters *9*, 2816-2825. <https://doi.org/10.1021/acsenergylett.4c01009>.

3. Zhang, M., Yu, P., Xiong, K., Wang, Y., Liu, Y., and Liang, Y. (2022). Construction of Mixed Ionic-Electronic Conducting Scaffolds in Zn Powder: A Scalable Route to Dendrite-Free and Flexible Zn Anodes. Advanced Materials *34*, 2200860. <https://doi.org/10.1002/adma.202200860>.

4. Zhao, X., Gao, Y., Cao, Q., Bu, F., Pu, J., Wang, Y., and Guan, C. (2023). A High-Capacity Gradient Zn Powder Anode for Flexible Zn-Ion Batteries. Advanced Energy Materials *13*, 2301741. <https://doi.org/10.1002/aenm.202301741>.

5. Yu, J., Yi, Z., Yan, X., Chen, R., Tan, S., Li, P., Zhang, T., Zhang, H., Liang, J., and Hou, F. (2024). Deeply Discharged, Quiescently Stable, and Long-Life Zn Anode by Spontaneous SEI Formation. Small *20*, 2402055. <https://doi.org/10.1002/smll.202402055>.

6. Yang, Z., Zhang, Q., Li, W., Xie, C., Wu, T., Hu, C., Tang, Y., and Wang, H. (2023). A Semi-solid Zinc Powder-based Slurry Anode for Advanced Aqueous Zinc-ion Batteries. Angewandte Chemie International Edition *62*, e202215306. <https://doi.org/10.1002/anie.202215306>.

7. Wu, J.-C., Shen, X., Zhou, H., Li, X., Gao, H., Ge, J., Xu, T., and Zhou, H. (2024). Zn-In Alloying Powder Solvent Free Electrode Toward High-Load Ampere-Hour Aqueous Zn-Mn Secondary Batteries. Small *20*, 2308541. <https://doi.org/10.1002/smll.202308541>.

8. Cao, C., Du, W., Li, C.C., Ye, M., Zhang, Y., Tang, Y., and Liu, X. (2023). Designing multidimensional hydration inhibitor towards the long cycling performance of zinc powder anode. Journal of Materials Chemistry A *11*, 14345-14355. <https://doi.org/10.1039/d3ta02154h>.

9. Xu, Z., Li, Y., Li, G., Zhang, H., and Wang, X. (2023). Reversible zinc powder anode via crystal facet engineering. Matter *6*, 3075-3086. <https://doi.org/10.1016/j.matt.2023.06.045>.

10. Shi, G., Peng, X., Zeng, J., Zhong, L., Sun, Y., Yang, W., Zhong, Y.L., Zhu, Y., Zou, R., Admassie, S., et al. (2023). A Liquid Metal Microdroplets Initialized Hemicellulose Composite for 3D Printing Anode Host in Zn-Ion Battery. Advanced Materials *35*, 2300109. <https://doi.org/10.1002/adma.202300109>.

11. Zou, J., Zeng, Z., Wang, C., Zhu, X., Zhang, J., Lan, H., Li, L., Yu, Y., Wang, H., Zhu, X., et al. (2023). Ultraconformal Horizontal Zinc Deposition toward Dendrite-Free Anode. Small Structures *4*, 2200194. <https://doi.org/10.1002/sstr.202200194>.

12. Kidanu, W.G., Yang, H., Park, S., Hur, J., and Kim, I.T. (2022). Room-Temperature Liquid-Metal Coated Zn Electrode for Long Life Cycle Aqueous Rechargeable Zn-Ion Batteries. Batteries *8*, 208. <https://doi.org/10.3390/batteries8110208>.

13. Guo, X., Bi, X., Song, W., Zheng, J., Wang, L., Shi, D., Sun, Y., Zhao, J., and Dai, H. (2025). Ultrafast fabrication of a stable three-dimensional-structured GaIn@Zn anode using ultrasound in a room temperature liquid metal solvent for enhanced performance in zinc-ion batteries. Journal of Alloys and Compounds *1035*, 181502. <https://doi.org/10.1016/j.jallcom.2025.181502>.

14. Chen, H., Guo, Z., Wang, H., Huang, W., Pan, F., and Wang, Z. (2023). A liquid metal interlayer for boosted charge transfer and dendrite-free deposition toward high-performance Zn anodes. Energy Storage Materials *54*, 563-569. <https://doi.org/10.1016/j.ensm.2022.11.013>.

15. Jia, H., Wang, Z., Dirican, M., Qiu, S., Chan, C.Y., Fu, S., Fei, B., and Zhang, X. (2021). A liquid metal assisted dendrite-free anode for high-performance Zn-ion batteries. Journal of Materials Chemistry A *9*, 5597-5605. <https://doi.org/10.1Zn039/D0TA11828A>.

16. Pu, J., Cao, Q., Gao, Y., Wang, Q., Geng, Z., Cao, L., Bu, F., Yang, N., and Guan, C. (2024). Liquid Metal-Based Stable and Stretchable Zn-Ion Battery for Electronic Textiles. Advanced Materials *36*, 2305812. <https://doi.org/10.1002/adma.202305812>.

17. Li, X., Li, Q., Hou, Y., Yang, Q., Chen, Z., Huang, Z., Liang, G., Zhao, Y., Ma, L., Li, M., et al. (2021). Toward a Practical Zn Powder Anode: Ti_3_C_2_T_x_ MXene as a Lattice-Match Electrons/Ions Redistributor. ACS Nano 15, 14631-14642. https://doi.org/10.1021/acsnano.1c04354.

18. Sun, Y., Jin, Y., and Huang, J. (2025). Passivated Zn Powders as Metal Anode. Advanced Materials Interfaces 12, 2400643. https://doi.org/10.1002/admi.202400643.
