# Supplementary figures and images for: Dynamically Modified Flexible Zn Powder Anodes with Stable Performance at High Rate and High Zn Utilization
Source: Adv Sci (Weinh). 2026 Jun 15:e75967. Online ahead of print. doi: 10.1002/advs.75967 (PMC13336622; doi:10.1002/advs.75967)

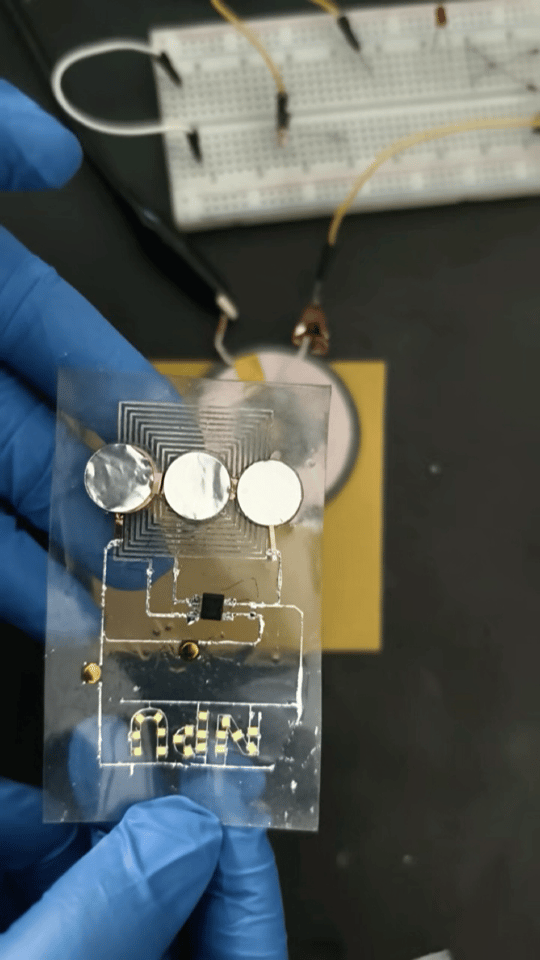

Supplement: Supplementary file 2 — Supporting File 2: advs75967‐sup‐0002‐MovieS1.GIF. [file ADVS-9999-e75967-s002.GIF]

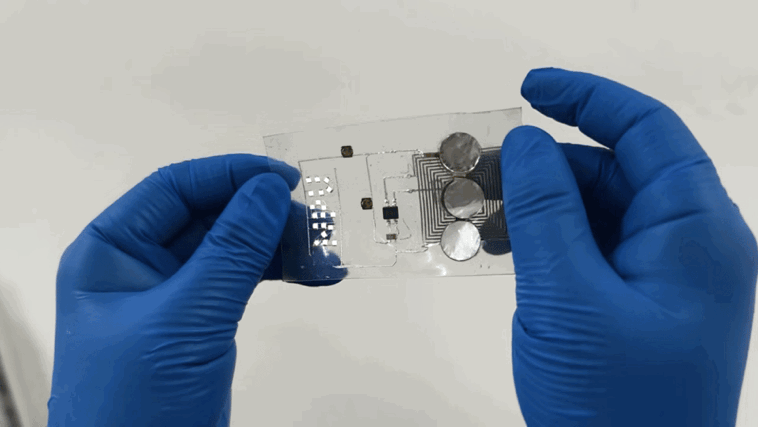

Supplement: Supplementary file 3 — Supporting File 3: advs75967‐sup‐0003‐MovieS2.GIF. [file ADVS-9999-e75967-s001.GIF]
